# Supplementary material for: Evaluation for Potential Drug–Drug Interaction of MT921 Using In Vitro Studies and Physiologically–Based Pharmacokinetic Models
Source: Pharmaceuticals (Basel). 2021 Jul 7;14(7):654. doi: 10.3390/ph14070654 (PMC8308925; doi:10.3390/ph14070654)
Supplement: Supplementary file 1 [file pharmaceuticals-14-00654-s001.zip › pharmaceuticals-1265322-supplementary.pdf]

pharmaceuticals

# Supplementary Materials: Evaluation for Potential Drug-Drug Interaction of MT921 Using In Vitro Studies and Physiologically Based Pharmacokinetic Models

Hyo-jeong Ryu, Hyun-ki Moon, Junho Lee, Gi-hyeok Yang, Sung-yoon Yang, Hwi-yeol Yun, Jung-woo Chae, Won-ho Kang

**Table of contents**

1. Inhibition Studies ... 2
1.1. Inhibition of MT921 on Cytochrome P450 (CYP) Isoforms ... 2
1.2. Inhibition of MT921 on UDP-glucuronosyltransferase (UGT) Isoforms ... 2
2. Transporter Substrate Specificity Studies ... 3
2.1. Substrate Specificity of MT921 on the Bidirectional Transport Activities for MDR1, BCRP, MRP2, and BSEP ... 3
2.2. Substrate Specificity of MT921 for OATP2B1, OAT1, OCT1, OCT2, MATE1, and MATE2K ... 4
3. Transporter Inhibition Studies ... 5
3.1. Inhibitory Effects of MT921 on the Bidirectional Transport Activities MDR1, BCRP, MRP2, and BSEP ... 5
3.2. Inhibitory Effects of MT921 on the Transport Activities OATP2B1, OAT1, OCT1, OCT2, MATE1, and MATE2K ... 6
4. Induction Studies ... 7
4.1. Viability of Hepatocytes Treatment with MT921 ... 7
4.2. Effects of MT921 on the Expression of CYPs, UGTs, and Transporters ... 8
5. Physiologically Based Pharmacokinetic (PBPK) Modeling of MT921 ... 10
5.1. Clinical Studies ... 10
5.2. Drug-Dependent Parameters ... 11
5.3. Profile ... 11
5.3.1. Semilogarithmic Plots of Population Predictions ... 11
5.3.2. Linear Plots of Population Predictions ... 12
5.4. MT921 PBPK Model Evaluation ... 13
5.4.1. Plasma Concentration Goodness-of-Fit Plot ... 13
5.4.2. Mean Relative Deviation (MRD) of Predicted Plasma Concentrations ... 13
5.4.3. Predicted and Observed Pharmacokinetic (PK) Parameter Values with Mean GMFE Values ... 13
5.4.4. Sensitivity Analysis ... 14
6. PBPK Modeling of Amlodipine ... 14
6.1. Clinical Studies ... 14
6.2. Drug-Dependent Parameters ... 15
6.3. Profile ... 15
6.3.1. Semilogarithmic Plots – Plasma – Population Predictions ... 15
6.3.2. Linear Plots – Plasma – Population Predictions ... 18
6.4. Amlodipine PBPK Model Evaluation ... 20
6.4.1. Plasma Concentration Goodness-of-Fit Plot ... 20
6.4.2. Mean Relative Deviation (MRD) of Predicted Plasma Concentrations ... 21
6.4.3. Predicted and Observed Pharmacokinetic (PK) Parameter Values with Mean GMFE Values ... 22
6.4.4. Sensitivity Analysis ... 22
7. Prediction of DDI Potential ... 23
7.1. Profile ... 23
7.1.1. Linear Graph – Comparing MT921 Alone Concentration and Population Simulation of MT921 Co-Administration. ... 23
7.1.2. Semilogarithmic Graph – Comparing MT921 Alone Concentration and Population Simulation of MT921 Co-Administration ... 24

1. **Inhibition Studies**

*1.1. Inhibition of MT921 on Cytochrome P450 (CYP) Isoforms*

The inhibitory effects of MT921 on the nine CYP isoforms (1A2, 2A6, 2B6, 2C8, 2C9, 2C19, 2D6, 2E1, 3A) in pooled human liver microsomal incubation system. The inhibition effect of MT921 were measured with nine substrates cocktail (phenacetin for 1A2, coumarin for 2A6, bupropion for 2B6, rosiglitazone for 2C8, diclofenac for 2C9, S-mephenytoin for 2C19, dextromethorphan for 2D6, chlorzoxazone for 2E1, midazolam or testosterone for 3A) and serial concentration of MT921 (0~100 μM) with enzymatic reaction time of 20 min [1]. From the microsomal incubation system used in this experiment, MT921 did not show inhibitory potential on nine CYP isoforms ($IC_{50}$ > 100 μM) (Table S1). The MT921 seems to have no significant inhibitory interaction potential on CYP-mediated enzyme activity *in vitro*.

**Table S1.** Summary of inhibitory potential of MT921 on nine CYP isoform catalytic activities in pooled human liver microsomal incubation system.

| **CYP isozyme** | **Metabolite** | **MT921 $IC_{50}$ (μM)** | **Positive control $IC_{50}$ (μM)*** |
|---|---|---|---|
| 1A2 | Phenacetin O-deethylation | > 100 | 0.79 (Furafylline) |
| 2A6 | Coumarin 7-hydroxylation | > 100 | 0.14 (8-Methoxypsoralen) |
| 2B6 | Bupropion 6-hydroxylation | > 100 | 1.2 (Ticlopidine) |
| 2C8 | Rosiglitazone hydroxylation | > 100 | 8.47 (Quercetin) |
| 2C9 | Diclofenac hydroxylation | > 100 | 1.22 (Sulfaphenazole) |
| 2C19 | S-Mephenytoin 4′-hydroxylation | > 100 | 0.44 (S-benzylnirvanol) |
| 2D6 | Dextromethorphan O-demethylation | > 100 | 0.06 (Quinidine) |
| 2E1 | Chlorzoxazone 6-hydroxylation | > 100 | 16.7 (Diethyldithio-carbamate) |
| 3A | Midazolam 1′-hydroxylation | > 100 | 0.095 (Ketoconazole) |
| 3A | Testosterone 6β-hydroxylation | > 100 | 0.053 (Ketoconazole) |

*The positive control results was in-house data, similar to the reference values [1]. All tests were assayed in triplicate. CYP, cytochrome P450; IC50, drug concentration at half of inhibition.

*1.2. Inhibition of MT921 on UDP-glucuronosyltransferase (UGT) Isoforms*

The inhibitory effects of MT921 on the six UGT isoforms (1A1, 1A3, 1A4, 1A6, 1A9, 2B7) in pooled human liver microsomal incubation system. The inhibition effect of MT921 were measured with six substrates cocktail (β-estradiol for 1A1, chenodeoxycholic acid for 1A3, trifluoperazine for 1A4, 4-hydroxyindole for 1A6, propofol for 1A9, naloxone for 2B7) and serial concentration of MT921 (0~100 μM) with enzymatic reaction time of 60 min [2]. From the microsomal incubation system used in this experiment, MT921 did not

show inhibitory potential on nine UGT isoforms ($IC_{50}$ > 100 μM) (Table S2). The MT921 seems to have no significant inhibitory interaction potential on UGT-mediated enzyme activity *in vitro*.

**Table S2.** Summary of inhibitory potential of MT921 on six UGT isoform catalytic activities in pooled human liver microsomal incubation system.

| UGT isozyme | Metabolite | MT921 $IC_{50}$ (μM) | Positive control $IC_{50}$ (μM)* |
|---|---|---|---|
| 1A1 | Estradiol 3-glucuronidation | > 100 | 0.29 (Atazanavir) |
| 1A3 | Chenodeoxycholic acid glucuronidation | > 100 | 14.1 (Glycyrrhetic acid) |
| 1A4 | Trifluoperazine glucuronidation | > 100 | 2.1 (Hecogenin) |
| 1A6 | 4-hydroxyindole glucuronidation | > 100 | 63.8 (Troglitazone) |
| 1A9 | Propofol glucuronidation | > 100 | 0.28 (Niflumic acid) |
| 2B7 | Naloxone-3-glucuronidation | > 100 | 27.3 (Efavirenz) |

*The positive control results was in-house data, similar to the reference values [2]. All tests were assayed in triplicate. UGT, UDP-glucuronosyltransferase; IC50, drug concentration at half of inhibition.

## 2. Transporter Substrate Specificity Studies

### *2.1. Substrate Specificity of MT921 on the Bidirectional Transport Activities for MDR1, BCRP, MRP2, and BSEP*

The substrate specificity of MT921 for efflux transporters were determined by measuring bidirectional transport ratio (efflux ratio) of MT921 (10 and 100 μM) in MDR1-, BCRP-, MRP2-, and BSEP-overexpressing MDCKII cells. To confirm of our expressing cells systems, there was confirmed that the efflux ratio of the positive control substrate into transporter-expressing cells calculated from apparent permeability of basal to apical direction transport rate divided by the apical to basal direction transport rate. The efflux ratio of probe substrates (1 μM rhodamine 123 for MDR1, 10 μM [$^3$H]estrone-3-sulfate for BCRP, 10 μM [$^3$H]vinblastine for MRP2, 0.8 μM [$^3$H]taurocholate for BSEP) in transporter-expressing cells were greatly increased and were decreased in presence of representative inhibitors (40 μM cyclosporine A for MDR1 and MRP2, 20 μM Ko143 for BCRP, 50 μM paclitaxel for BSEP) (4.38-fold increase in the efflux ratio of rhodamine 123 for MDR1; 3.91-fold increase in the efflux ratio of [$^3$H]estrone-sulfate for BCRP; 6.37-fold increase in the efflux ratio of [$^3$H]vinblastine for MRP2; 3.35-fold increase in the efflux ratio of [$^3$H]taurocholate for BSEP) [3, 4]. The efflux ratio of MT921 at two different concentrations (10 and 100 μM) were less than 2-fold in MDR1, BCRP, MRP2, and BSEP, respectively (Table S3). These results indicated that MT921 were determined not to be a substrate for MDR1, BCRP, MRP2 and BSEP based on less than 2-fold of efflux ratio cutoff value in U.S. Food and Drug Administration (FDA) guideline [5].

**Table S3.** Apical to Basal (A to B) and Basal to Apical (B to A) directional apparent permeability of MT921 (10 and 100 μM) in MDCKII-MDR1, -BCRP, -MRP2-, and BSEP.

| Concentration (μM) | Transporter | Apparent permeability (× 10⁻⁶ cm/s) | Efflux ratio (Apparent permeability B to A / Apparent permeability A to B) |
|---|---|---|---|
| 10 | MDCKII-MDR1 A to B | 21.8 ± 0.43 | 1.00 |
| | MDCKII-MDR1 B to A | 23.6 ± 0.95 | 1.08 |
| 100 | MDCKII-MDR1 A to B | 22.7 ± 2.31 | 1.00 |
| | MDCKII-MDR1 B to A | 20.2 ± 5.31 | 0.89 |
| 10 | MDCKII-BCRP A to B | 19.1 ± 2.19 | 1.00 |
| | MDCKII-BCRP B to A | 20.3 ± 1.16 | 1.06 |
| 100 | MDCKII-BCRP A to B | 22.5 ± 0.71 | 1.00 |
| | MDCKII-BCRP B to A | 21.6 ± 3.03 | 0.96 |
| 10 | MDCKII-MRP2 A to B | 12.6 ± 1.52 | 1.00 |
| | MDCKII-MRP2 B to A | 14.4 ± 0.72 | 1.14 |
| 100 | MDCKII-MRP2 A to B | 15.5 ± 1.10 | 1.00 |
| | MDCKII-MRP2 B to A | 15.7 ± 2.24 | 1.01 |
| 10 | MDCKII-BSEP A to B | 15.1 ± 1.80 | 1.00 |
| | MDCKII-BSEP B to A | 17.7 ± 1.08 | 1.17 |
| 100 | MDCKII-BSEP A to B | 18.7 ± 0.45 | 1.00 |
| | MDCKII-BSEP B to A | 17.3 ± 2.43 | 0.93 |

Each data represents mean ± standard deviation from triplicate determinations. MDCK, Madin-Darby canine kidney; MDR, multidrug resistance protein; BCRP, breast cancer resistance protein; MRP, multidrug resistance-associated protein; BSEP, bile salt export pump.

### *2.2. Substrate Specificity of MT921 for OATP2B1, OAT1, OCT1, OCT2, MATE1, and MATE2K*

The uptake rates of MT921 (10 and 100 μM) were examined in OATP2B1-, OAT1-, OCT1-, OCT2-, MATE1-, and MATE2K-overexpressing HEK 293 cells and mock cells. To confirm of our expressing cells systems, there was confirmed that the uptake rates of the positive control substrate into transporter-expressing cells and mock cells. The uptake rates of probe substrates (0.022 μM [$^3$H]estrone-3-sulfate for OATP2B1, 0.930 μM [$^3$H]*para*-aminohippuric acid for OAT1, 4 μM [$^{14}$C]Metformin for OCT1, OCT2, MATE1, and MATE2K) in transporter-expressing cells were greatly increased compared to that in mock cells and were decreased in presence of representative inhibitors (30 μM bromsulphthalein for OATP2B1, 100 μM probenecid for OAT1, 100 μM verapamil for OCT1, 200 μM verapamil for OCT2, 50 μM quinidine for MATE1 and MATE2K) (13.0-fold increase in [$^3$H]estrone-3-sulfate for OATP2B1; 127.6-fold increase in [$^3$H]*para*-aminohippuric acid for OAT1; 4.8-, 8.0-, 18.3-, and 4.2-fold increase in [$^{14}$C]Metformin for OCT1, OCT2, MATE1, and MATE2K, respectively) [6-9]. The uptake ratio of MT921 at 10 and 100 μM were less than or equal to 2-fold in OATP2B1, OAT1, OCT1, OCT2, MATE1, and MATE2K, respectively (Table S4). These results indicated that MT921 were determined not to be a substrate for these transporters based on less than 2-fold of uptake ratio cutoff value in U.S. FDA guideline [5].

**Table S4.** The uptake transport rate of MT921 (10 and 100 μM) in HEK 293-OATP2B1, -OAT1, -OCT1, -OCT2, -MATE1, and –MATE2K and mock cells.

| Concentration (μM) | Transporter | Uptake (pmol/mg protein/min) | Uptake ratio (transporter vs mock) |
|---|---|---|---|
| 10 | Mock | 7.61 ± 0.91 | 1.00 |
| | HEK 293-OATP2B1 | 12.52 ± 0.78 | 1.65 |

| | | | |
|---|---|---|---|
| 100 | Mock | 72.15 ± 3.91 | 1.00 |
| | HEK 293-OATP2B1 | 153.66 ± 10.07 | 2.13 |
| 10 | Mock | 7.61 ± 0.91 | 1.00 |
| | HEK 293-OAT1 | 11.82 ± 0.93 | 1.55 |
| 100 | Mock | 72.15 ± 3.91 | 1.00 |
| | HEK 293-OAT1 | 153.66 ± 10.07 | 2.15 |
| 10 | Mock | 4.85 ± 0.55 | 1.00 |
| | HEK 293-OCT1 | 3.92 ± 0.23 | 0.81 |
| 100 | Mock | 57.16 ± 0.67 | 1.00 |
| | HEK 293-OCT1 | 39.79 ± 3.28 | 0.70 |
| 10 | Mock | 4.85 ± 0.55 | 1.00 |
| | HEK 293-OCT2 | 6.67 ± 0.22 | 1.38 |
| 100 | Mock | 57.16 ± 0.67 | 1.00 |
| | HEK 293-OCT2 | 67.10 ± 0.95 | 1.17 |
| 10 | Mock | 6.78 ± 0.96 | 1.00 |
| | HEK 293-MATE1 | 7.48 ± 0.74 | 1.10 |
| 100 | Mock | 68.16 ± 3.49 | 1.00 |
| | HEK 293- MATE1 | 86.20 ± 5.62 | 1.26 |
| 10 | Mock | 6.78 ± 0.96 | 1.00 |
| | HEK 293-MATE2K | 9.40 ± 0.93 | 1.39 |
| 100 | Mock | 68.16 ± 3.49 | 1.00 |
| | HEK 293- MATE2K | 94.23 ± 11.32 | 1.38 |

Each data represents mean ± standard deviation from triplicate determinations. HEK, human embryonic kidney; OATP, organic anion transporting polypeptide; OAT, organic anion transporter; OCT, organic cation transporter; MATE, multidrug and toxin extrusion.

## 3. Transporter Inhibition Studies

### *3.1. Inhibitory Effects of MT921 on the Bidirectional Transport Activities MDR1, BCRP, MRP2, and BSEP*

The inhibitory effects of MT921 on the MDR1, BCRP, MRP2, and BSEP transport activities were evaluated using MDCKII-MDR1, -BCRP, - MRP2, and –BSEP stable cells. To confirm of MDR1-, BCRP-expressing cells systems, there was confirmed that the efflux ratio of the positive control substrate into transporter-expressing cells calculated from apparent permeability of basal to apical direction transport rate divided by the apical to basal direction transport rate. The efflux ratio of probe substrates (10 μM [$^3$H]digoxin for MDR1, 10 μM [$^3$H]estrone-3-sulfate for BCRP) in transporter-expressing cells were greatly increased and were decreased in presence of representative inhibitors (40 μM cyclosporine A for MDR1, 20 μM Ko143 for BCRP) (4.62-fold increase in the efflux ratio of [$^3$H]digoxin for MDR1; 4.64-fold increase in the efflux ratio of [$^3$H]estrone-3-sulfate for BCRP) [3, 4]. MT921 (1, 10, and 100 μM) did not inhibit bidirectional transport activity of both MDR1 and BCRP up to 100 μM ($IC_{50}$ > 100 μM) (Figure S1).

In the case of MRP2, and BSEP, intracellular accumulation of probe substrates (0.5 μM calcein AM for MRP2, 10 μM [$^3$H]taurocholate for BSEP) in MDCKII-MRP2 and-BSEP cells were maximally accumulated in the presence of representative inhibitors (40 μM cyclosporine A for MRP2, 100 μM paclitaxel for BSEP) because MRP2 and BSEP could not be pumped out their substrate (calcein AM and taurocholic acid) in the presence of inhibitor, which is considered maximum inhibition as positive controls [3, 4]. MT921 (0.5~100 μM) did not inhibit transport activity of both MRP2 and BSEP up to 100 μM ($IC_{50}$ > 100 μM) (Figure S2).

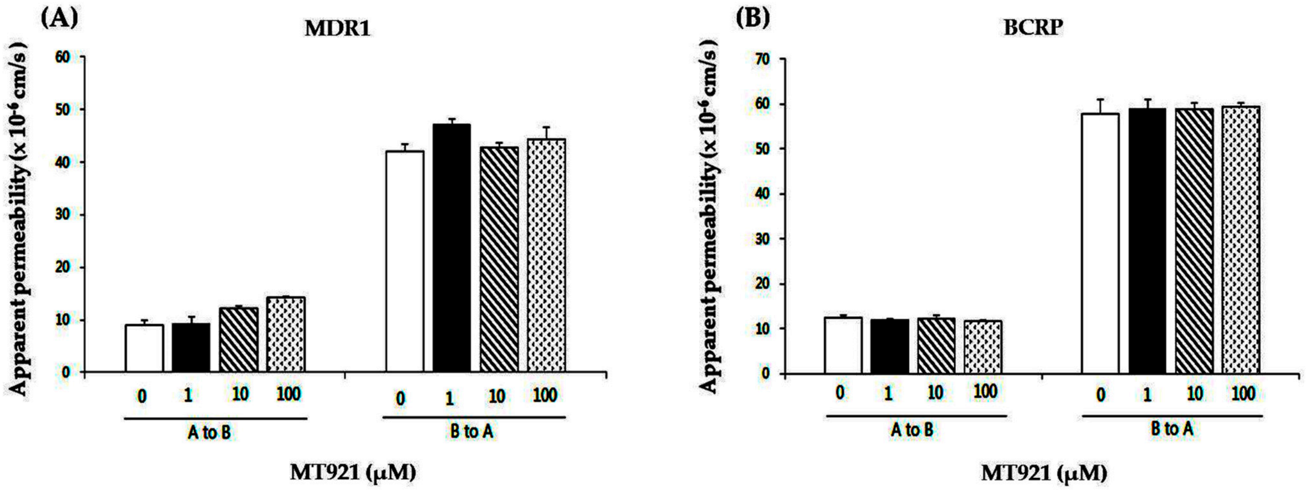


**Figure S1.** Inhibitory effects of MT921 (1, 10, and 100 μM) on bidirectional transport activity using probe substrates ([3H]digoxin for MDR1, [3H]estrone-3-sulfate for BCRP) in MDCKII (**A**) –MDR1; (**B**) –BCRP. Each bar represents mean ± standard deviation from triplicate determinations. MDCK, Madin-Darby canine kidney; MDR, multidrug resistance protein; BCRP, breast cancer resistance protein.

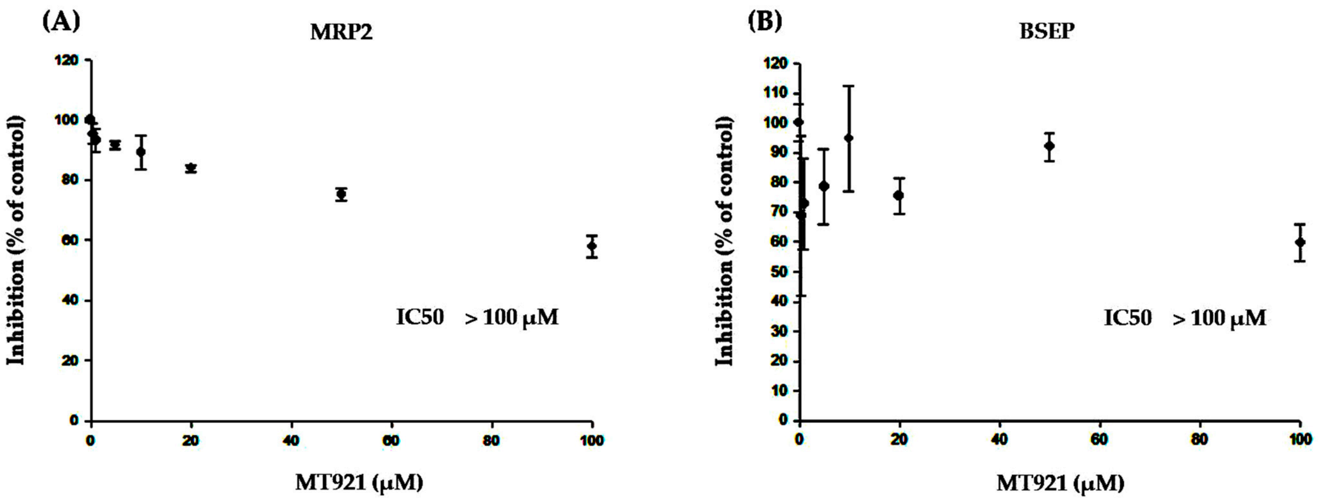


**Figure S2.** Inhibitory effects of MT921 (0.5~100 μM) on intracellular accumulation of probe substrates (calcein AM for MRP2, [3H]taurocholate for BSEP) in MDCKII (**A**) –MRP2; (**B**) –BSEP. Each bar represents mean ± standard deviation from triplicate determinations. $IC_{50}$, drug concentration at half of inhibition. MDCK, Madin-Darby canine kidney; MRP, multidrug resistance-associated protein; BSEP, bile salt export pump.

### *3.2. Inhibitory Effects of MT921 on the Transport Activities OATP2B1, OAT1, OCT1, OCT2, MATE1, and MATE2K*

The inhibitory effects of MT921 on the OATP2B1, OAT1, OCT1, OCT2, MATE1, and MATE2K transport activities were evaluated using HEK 293-OATP2B1, -OAT1, -OCT1, -OCT2, -MATE1, and -MATE2K stable cells. The uptake rate were measured uptake of probe substrates (0.022 μM [3H]estrone-3-sulfate for OATP2B1, 0.930 μM [3H]*para*-aminohippuric acid for OAT1, 4 μM [14C]Metformin for OCT1, OCT2, MATE1, and MATE2K) into transporter-expressing cells and mock cells in the absence and presence of MT921 or representative inhibitors (30 μM bromsulphthalein for OATP2B1, 100 μM probenecid for

OAT1, 100 μM verapamil for OCT1, 200 μM verapamil for OCT2, 50 μM quinidine for MATE1 and MATE2K) [6-9]. The positive inhibitors strongly inhibited the transport activity of OATP2B1, OAT1, OCT1, OCT2, MATE1, and MATE2K that the *in vitro* test systems were capable of detecting inhibitors of those transporters. Over the concentration range tested (0.5~100 μM), MT921 did not showed the inhibition effect on OATP2B1, OAT1, OCT1, OCT2, MATE1 and MATE2K (Figure S3).

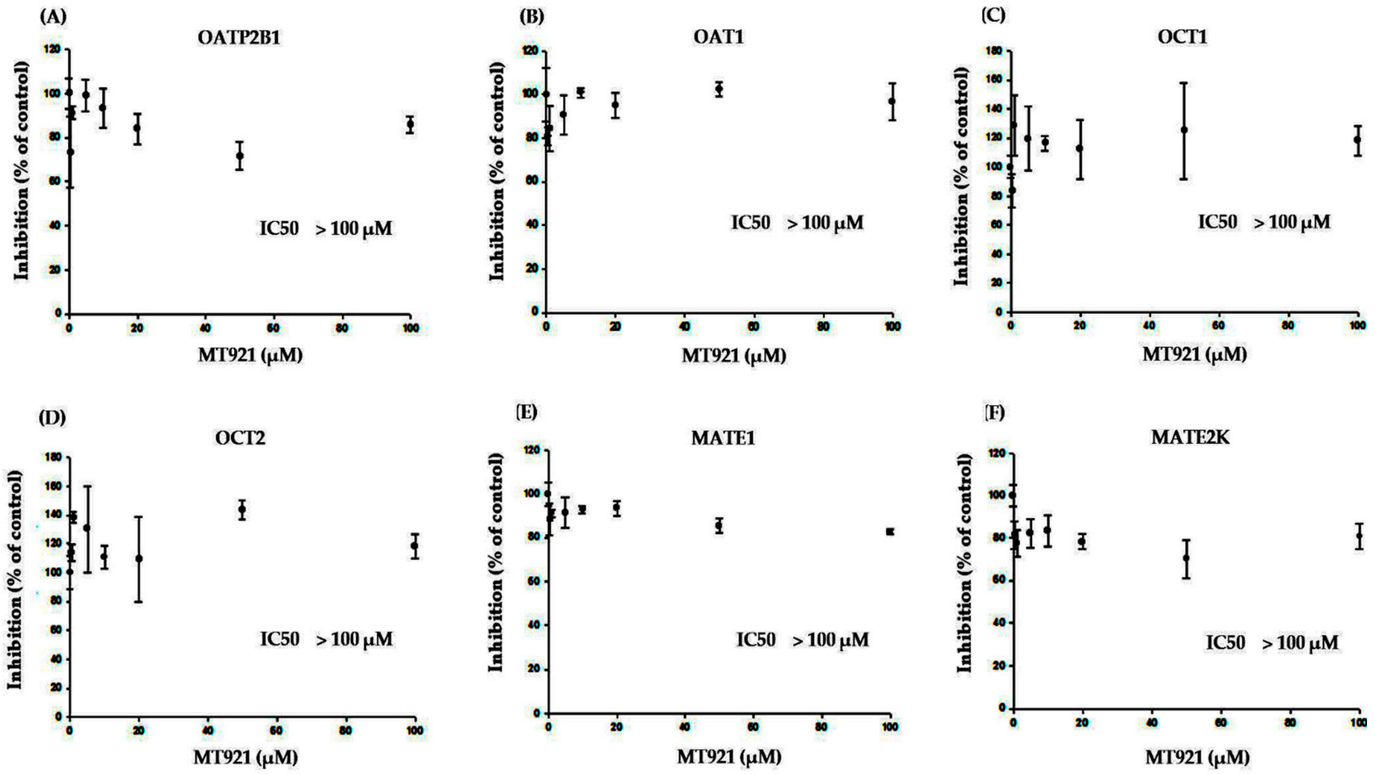


**Figure S3.** Concentration-dependent inhibition effect of MT921 (0.5~100 μM) on (**A**) 0.022 μM [$^3$H]estrone-3-sulfate in HEK 293-OATP2B1; (**B**) 0.930 μM [$^3$H]*para*-aminohippuric acid in HEK 293-OAT1; (**C**) 4 μM [$^{14}$C]Metformin in HEK 293-OCT1; (**D**) 4 μM [$^{14}$C]Metformin in HEK 293- OCT2; (**E**) 4 μM [$^{14}$C]Metformin in HEK 293-MATE1; (**F**) 4 μM [$^{14}$C]Metformin in HEK 293-MATE2K. Each bar represents mean ± standard deviation from triplicate determinations. HEK, human embryonic kidney; OATP, organic anion transporting polypeptide; OAT, organic anion transporter; OCT, organic cation transporter; MATE, multidrug and toxin extrusion; $IC_{50,}$ drug concentration at half of inhibition.

## 4. Induction Studies

### *4.1. Viability of Hepatocytes Treatment with MT921*

We evaluate the cell toxicity effect of MT921 (0.5~100 μM) for 5 min, 30 min, 60 min, 24 hours, and 48 hours on hepatocytes from one donor using the ATP-based CellTiter-Glo Luminescent assay kit (Promega, Madison, WI, USA) [10]. The batch of cryopreserved human hepatocytes (Catalog No. 454551, Lot No. 349) was purchased from Corning-Gentest (Tewksbury, MA, USA). The cryopreserved hepatocytes obtained from commercial sources in cell viability study were characterized by the following; african american man (ages 44), post-thaw cell viability was 86 %.

Hepatocytes treated with MT921 showed above 90 % viability up to 100 μM for 48 hours (Table S5). These results suggested MT921 did not showed cytotoxicity effect on hepatocytes in this experiment condition.

**Table S5.** Cell viability of MT921 (0~100 μM) in cryopreserved human hepatocytes.

| Incubation time | Concentration (μM) | Cell viability (%) |
|---|---|---|
| 5 min | 0 | 100 ± 1.74 |
| | 1 | 92 ± 8.61 |
| | 5 | 90 ± 1.25 |
| | 10 | 90 ± 8.40 |
| | 20 | 94 ± 14.35 |
| | 50 | 96 ± 7.46 |
| | 100 | 93 ± 0.10 |
| 30 min | 0 | 100 ± 18.17 |
| | 1 | 87 ± 5.68 |
| | 5 | 91 ± 3.20 |
| | 10 | 98 ± 3.37 |
| | 20 | 86 ± 1.61 |
| | 50 | 94 ± 2.28 |
| | 100 | 92 ± 0.20 |
| 60 min | 0 | 100 ± 4.19 |
| | 1 | 90 ± 10.49 |
| | 5 | 102 ± 0.07 |
| | 10 | 97 ± 3.56 |
| | 20 | 99 ± 11.68 |
| | 50 | 106 ± 8.45 |
| | 100 | 104 ± 6.74 |
| 24 hours | 0 | 100 ± 6.73 |
| | 1 | 98 ± 8.48 |
| | 5 | 95 ± 5.35 |
| | 10 | 101 ± 7.93 |
| | 20 | 108 ± 0.79 |
| | 50 | 106 ± 2.69 |
| | 100 | 97 ± 0.75 |
| 48 hours | 0 | 100 ± 2.40 |
| | 1 | 96 ± 8.56 |
| | 5 | 101 ± 1.12 |
| | 10 | 96 ± 2.72 |
| | 20 | 95 ± 4.75 |
| | 50 | 96 ± 0.72 |
| | 100 | 101 ± 2.52 |

Each data represents mean ± standard deviation from duplicate determinations.

### *4.2. Effects of MT921 on the Expression of CYPs, UGTs, and Transporters*

To examine the induction of drug metabolizing enzymes and transporters by MT921, relative mRNA expression levels of CYPs (1A2, 2B6, 2C8, 2C9, 2C19, 3A), UGTs (1A1, 1A3, 1A4, 1A6, 1A9, 2B7), and drug transporters (MDR1, BCRP, MRP2, BSEP, OATP1B1, OCT1) were assessed in cryopreserved human hepatocytes of after the treatment of MT921 (1, 10 and 100 μM) for 48 hours. Three batches of cryopreserved human hepatocytes (Catalog No. 454551, Lot No. 302, 319, and 349) was purchased from Corning-Gentest (Tewksbury, MA, USA). The cryopreserved hepatocytes obtained from commercial sources in cell viability study were characterized by the following; two caucasian men and one african american (ages 33, 53, and 44), post-thaw cell viability were 81, 87, and 86 %, respectively. To confirm of our cell systems, rifampin 40 μM and omeprazole 100 μM were also treated as

positive controls for CYPs (2B6, 2C8, 2C9, 2C19, 3A, MDR1) and CYP1A2, respectively. The inducibility was decided by the 2-fold change in the mRNA expression level of respective enzymes and a response more than 20 % of the response of the positive control in the presence of the test compound based on U.S. FDA guideline [5]. The positive controls induced the expression levels of CYPs and MDR1 (i.e. 32.23-fold increase for CYP1A2, 4.60-fold increase for CYP2B6, 2.59-fold increase for CYP2C8, 2.47-fold increase for CYP2C9, 1.94-fold increase for CYP2C19, 39.74-fold increase for CYP3A4, 4.56-fold increase for CYP3A5, 2.55-fold increase for MDR1).

When measured inducibility of MT921 on drug metabolizing enzymes and transporters in cryopreserved human hepatocytes from three independent donors, MT921 (1, 10 and 100 μM) did not induced CYPs mRNA compared with vehicle controls. In addition, MT921 (1, 10 and 100 μM) did not induced mRNA expression of UGTs and mRNA expression of transporters compared with vehicle control (Table S6).

**Table S6.** Effects of MT921 (1, 10, and 100 μM) on CYPs, UGTs, and transporters mRNA expression in cryopreserved human hepatocytes.

| CYPs, UGTs, and transporters | Concentration (μM) | Fold induction of mRNA levels |
|---|---|---|
| CYP1A2 | Control | 1.00 ± 0.00 |
| | 1 | 0.78 ± 0.25 |
| | 10 | 0.81 ± 0.21 |
| | 100 | 0.80 ± 0.13 |
| CYP2B6 | Control | 1.00 ± 0.00 |
| | 1 | 1.07 ± 0.43 |
| | 10 | 1.17 ± 0.34 |
| | 100 | 1.09 ± 0.40 |
| CYP2C8 | Control | 1.00 ± 0.00 |
| | 1 | 1.08 ± 0.32 |
| | 10 | 1.06 ± 0.06 |
| | 100 | 1.20 ± 0.26 |
| CYP2C9 | Control | 1.00 ± 0.00 |
| | 1 | 0.76 ± 0.17 |
| | 10 | 0.89 ± 0.11 |
| | 100 | 0.85 ± 0.13 |
| CYP2C19 | Control | 1.00 ± 0.00 |
| | 1 | 1.18 ± 0.09 |
| | 10 | 1.35 ± 0.16 |
| | 100 | 1.51 ± 0.13 |
| CYP3A4 | Control | 1.00 ± 0.00 |
| | 1 | 0.93 ± 0.61 |
| | 10 | 1.01 ± 0.71 |
| | 100 | 1.19 ± 1.31 |
| CYP3A5 | Control | 1.00 ± 0.00 |
| | 1 | 0.80 ± 0.39 |
| | 10 | 0.78 ± 0.30 |
| | 100 | 0.97 ± 0.59 |
| UGT1A1 | Control | 1.00 ± 0.00 |
| | 1 | 0.94 ± 0.22 |
| | 10 | 1.09 ± 0.23 |
| | 100 | 1.24 ± 0.20 |
| UGT1A3 | Control | 1.00 ± 0.00 |
| | 1 | 1.03 ± 0.41 |

| | | |
|---|---|---|
| | 10 | 0.90 ± 0.30 |
| | 100 | 0.70 ± 0.31 |
| UGT1A4 | Control | 1.00 ± 0.00 |
| | 1 | 0.97 ± 0.12 |
| | 10 | 1.11 ± 0.16 |
| | 100 | 1.15 ± 0.08 |
| UGT1A6 | Control | 1.00 ± 0.00 |
| | 1 | 0.88 ± 0.10 |
| | 10 | 1.06 ± 0.21 |
| | 100 | 0.98 ± 0.27 |
| UGT1A9 | Control | 1.00 ± 0.00 |
| | 1 | 0.80 ± 0.06 |
| | 10 | 0.84 ± 0.17 |
| | 100 | 0.82 ± 0.12 |
| UGT2B7 | Control | 1.00 ± 0.00 |
| | 1 | 0.82 ± 0.14 |
| | 10 | 0.89 ± 0.18 |
| | 100 | 0.92 ± 0.08 |
| MDR1 | Control | 1.00 ± 0.00 |
| | 1 | 0.92 ± 0.34 |
| | 10 | 0.91 ± 0.24 |
| | 100 | 0.91 ± 0.40 |
| BCRP | Control | 1.00 ± 0.00 |
| | 1 | 0.91 ± 0.18 |
| | 10 | 0.89 ± 0.05 |
| | 100 | 0.97 ± 0.18 |
| MRP2 | Control | 1.00 ± 0.00 |
| | 1 | 0.88 ± 0.25 |
| | 10 | 0.95 ± 0.14 |
| | 100 | 0.92 ± 0.09 |
| BSEP | Control | 1.00 ± 0.00 |
| | 1 | 0.83 ± 0.26 |
| | 10 | 0.95 ± 0.30 |
| | 100 | 1.20 ± 0.34 |
| OATP1B1 | Control | 1.00 ± 0.00 |
| | 1 | 0.65 ± 0.14 |
| | 10 | 0.71 ± 0.01 |
| | 100 | 0.67 ± 0.18 |
| OCT1 | Control | 1.00 ± 0.00 |
| | 1 | 0.91 ± 0.42 |
| | 10 | 1.07 ± 0.10 |
| | 100 | 1.27 ± 0.34 |

Each data represents mean ± standard deviation from relative mRNA level of three individual hepatocytes. CYP, cytochrome P450; UGT, UDP-glucuronosyltransferase; MDR, multidrug resistance protein; BCRP, breast cancer resistance protein; MRP, multidrug resistance-associated protein; BSEP, bile salt export pump; OATP, organic anion transporting polypeptide; OCT, organic cation transporter.

## 5. Physiologically Based Pharmacokinetic (PBPK) Modeling of MT921

### 5.1. Clinical Studies

**Table S7.** MT921 clinical studies.

| Dose [mg] | Route | N | Age [years] | Weight [kg] | Height [cm] | Female [%] | Data set | Reference |
|---|---|---|---|---|---|---|---|---|
| 60 | sc, sd | 6 | 34,67 ± 12.04 | 81.3 ± 10.38 | 172.38 ± 5.71 | - | training | - |
| 120 | sc, sd | 6 | 32.67 ± 11.43 | 81,92 ± 8.14 | 175.67 ± 5.47 | - | training | - |
| 150 | sc, sd | 6 | 27.83 ± 4.36 | 83.23 ± 5.88 | 172 ± 4 | - | training | - |

sc, subcutaneous; sd, single dose; N, number of individuals in study; training, training data set for parameter optimization.

### 5.2. Drug-Dependent Parameters

**Table S8.** Drug-dependent parameters of the MT921 PBPK model.

| Parameter | Value | Unit | Source | Reference |
|---|---|---|---|---|
| MW | 408.57 | g/mol | - | - |
| Log P | 2.31 | Log units | Optimized | [11] |
| Fraction unbound | 51.84 | % | Optimized | [12] |
| Solubility | 280 | mg/l | - | - |
| Formulation | 41.10 | min | Optimized | |
| Total $CL_{hep}$ | 0.8 | 1/min | Optimized | [13] |
| ASBT $K_m$ | 34.90 | µM | Calculated | - |
| ASBT $K_{cat}$ | 11.40 | 1/m | Calculated | - |
| NTCP $K_m$ | 30.54 | µM | Calculated | - |
| NTCP $K_{cat}$ | 12.74 | 1/m | Calculated | - |
| OAT3 $K_m$ | 133.33 | µM | Calculated | - |
| OAT3 $K_{cat}$ | 10.22 | 1/m | Calculated | - |
| OATP1B3 $K_m$ | 27.44 | µM | Calculated | - |
| OATP1B3 $K_{cat}$ | 7.4 | 1/m | Calculated | - |
| GFR fraction | 1 | - | Assumed | - |
| EHC continuous fraction | 1 | - | Assumed | - |
| ASBT $K_i$ | 36.83 | µM | Calculated | Derived from $IC_{50}$ |
| NTCP $K_i$ | 46.16 | µM | Calculated | Derived from $IC_{50}$ |
| OAT3 $K_i$ | 68.64 | µM | Calculated | Derived from $IC_{50}$ |
| OATP1B3 $K_i$ | 38.20 | µM | Calculated | Derived from $IC_{50}$ |
| Partition coefficients | Schmitt | - | Calculated | [14] |
| Cellular perm. | PK-Sim standard | - | Calculated | [15] |
| Specific organ perm. | 5.27E-3 | cm/min | Optimized | - |
| Specific intest. perm. | 4.90E-7 | cm/min | Optimized | - |

MW, molecular weight; Log P, lipophilicity; $CL_{hep}$, total hepatic clearance; ASBT, apical sodium dependent bile acid transporter; NTCP, $Na^+$-taurocholate co-transporting polypeptide; OAT3, organic anion transporter 3; OATP1B3, organic anion transporting polypeptide 1B3; EHC, enterohepatic circulation; GFR, glomerular filtration rate; intest, intestinal; perm, permeability.

### 5.3. Profile

#### 5.3.1. Semilogarithmic Plots of Population Predictions

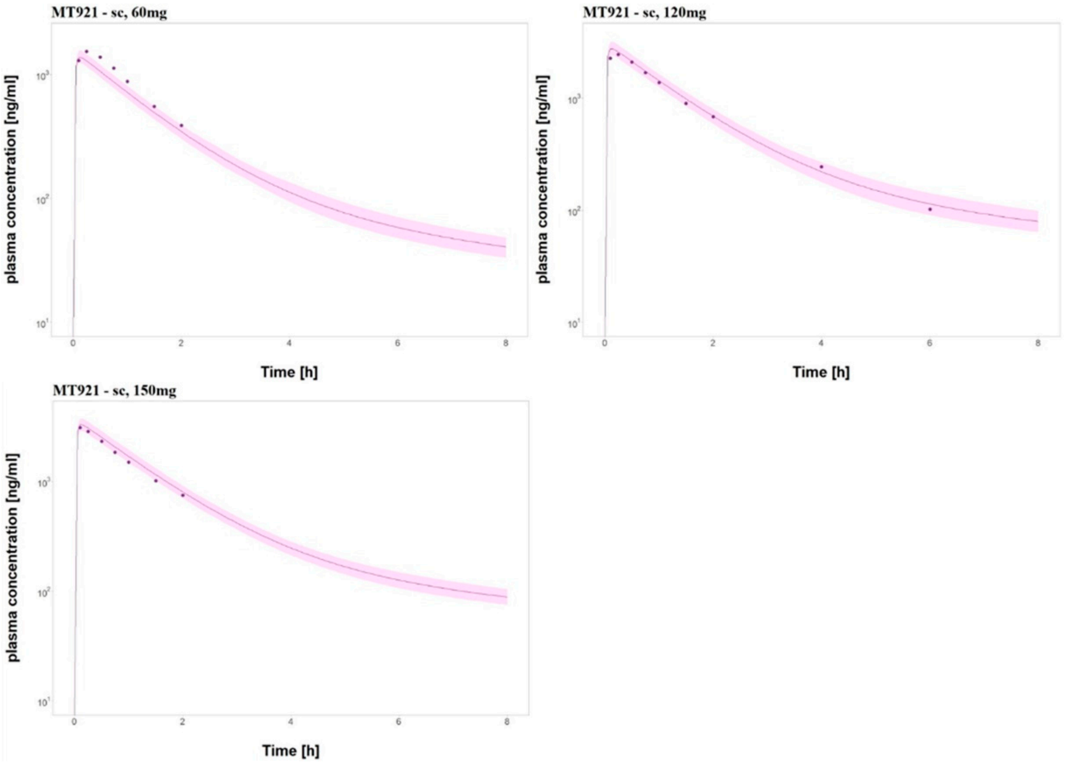


**Figure S4.** MT921 population plasma concentration-time profiles (semilogarithmic). Population predicted of MT921 plasma concentration-time profiles compared to observed data. Dot, observed data; Line, simulation arithmetic mean; shaded area, 95% confidence intervals of median predicted concentration.

### 5.3.2. Linear Plots of Population Predictions

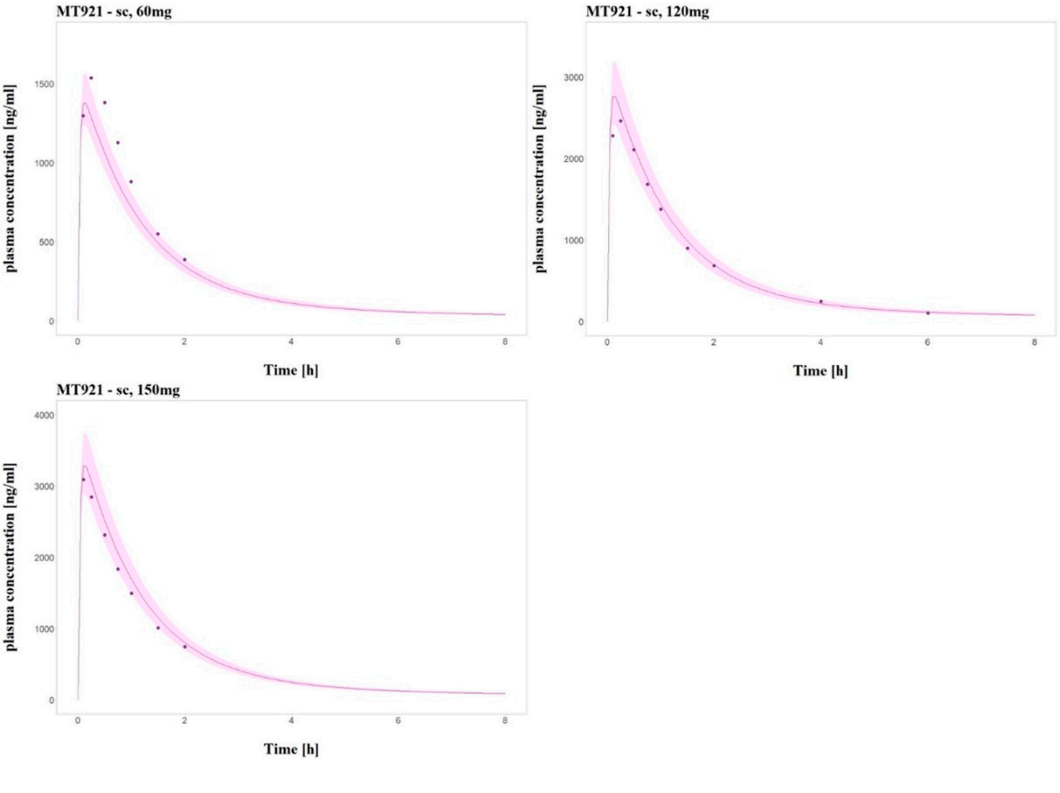

**Figure S5.** MT921 population plasma concentration-time profiles (linear). Population predictions of MT921 plasma concentration-time profiles compared to observed data. Dot, observed data; Line, simulation arithmetic mean; shaded area, 95% confidence intervals of median predicted concentration.

### *5.4. MT921 PBPK Model Evaluation*

#### 5.4.1. Plasma Concentration Goodness-of-Fit Plot

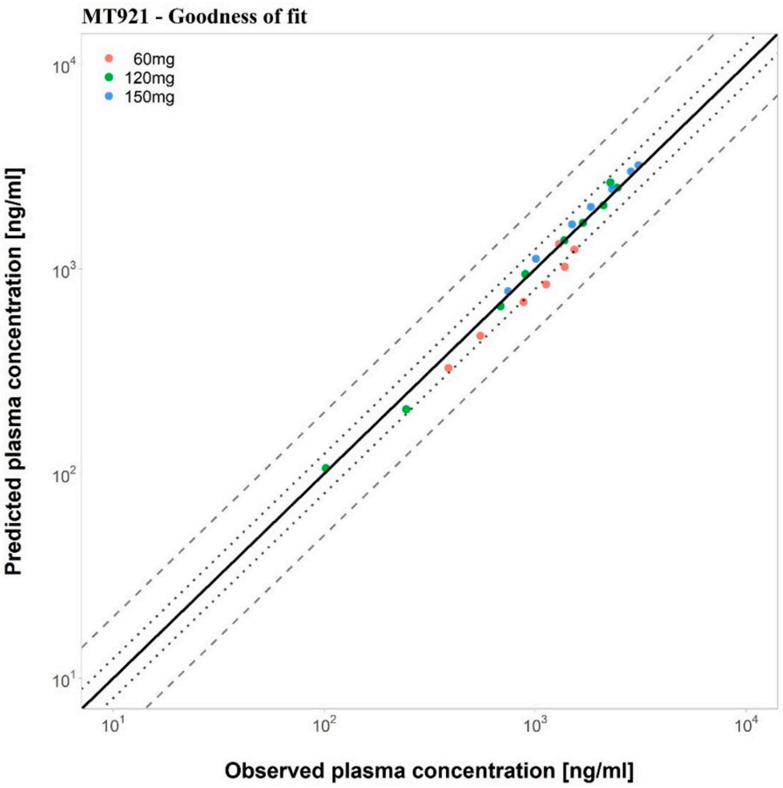


**Figure S6.** Goodness-of-fit plot of MT921. Predicted plasma concentration versus observed plasma concentration was compared in all clinical studies. The solid line means the line of identity. The dot lines are 1.25-fold, the dash lines is 2-fold deviation.

#### 5.4.2. Mean Relative Deviation (MRD) of Predicted Plasma Concentrations

**Table S9.** MRD values of predicted MT921 plasma concentration.

| Dose [mg] | Route | N | MRD | Reference |
|---|---|---|---|---|
| 60 | sc, sd | 6 | 1.24 | - |
| 120 | sc, sd | 6 | 1.08 | - |
| 150 | sc, sd | 6 | 1.08 | - |
| | | **MRD (range)** | **1.13 (1.08-1.24)** | |
| | | **MRD ≤2** | **3/3 studies** | |

sc, subcutaneous; sd, single dose; N, number of individuals in study; MRD, mean relative deviation.

#### 5.4.3. Predicted and Observed Pharmacokinetic (PK) Parameter Values with Mean GMFE Values

**Table S10.** Observed and predicted MT921 AUC and $C_{max}$ values with mean GMFE values.

| Dose [mg] | Route | N | AUCobs [ng/ml/h] | AUCpred [ng/ml/h] | Pred/Obs AUC | $C_{max}$ obs [ng/ml] | $C_{max}$ pred [ng/ml] | Pred/Obs $C_{max}$ | Reference |
|---|---|---|---|---|---|---|---|---|---|

| | | | | | | | | | |
|---|---|---|---|---|---|---|---|---|---|
| 60 | sc, sd | 6 | 1734.36 | 1474.98 | 0.85 | 1535.14 | 1328.91 | 0.86 | - |
| 120 | sc, sd | 6 | 3091.22 | 3540.17 | 1.14 | 2462.52 | 2659.14 | 1.07 | - |
| 150 | sc, sd | 6 | 4029.16 | 3989.46 | 0.99 | 3091.41 | 3215.87 | 1.04 | - |
| | | | **GMFE [range]** | | **1.10 (1.01-1.17)** | | | **1.09 (1.04 -1.16)** | |
| | | | **GMFE ≤2** | | **3/3 studies** | | | **3/3 studies** | |

$C_{max}$, peak plasma concentration; GMFE, geometric mean fold error; sc, subcutaneous; sd, single dose; N, number of individuals in study; obs, observed; pred, predicted.

5.4.4. Sensitivity Analysis

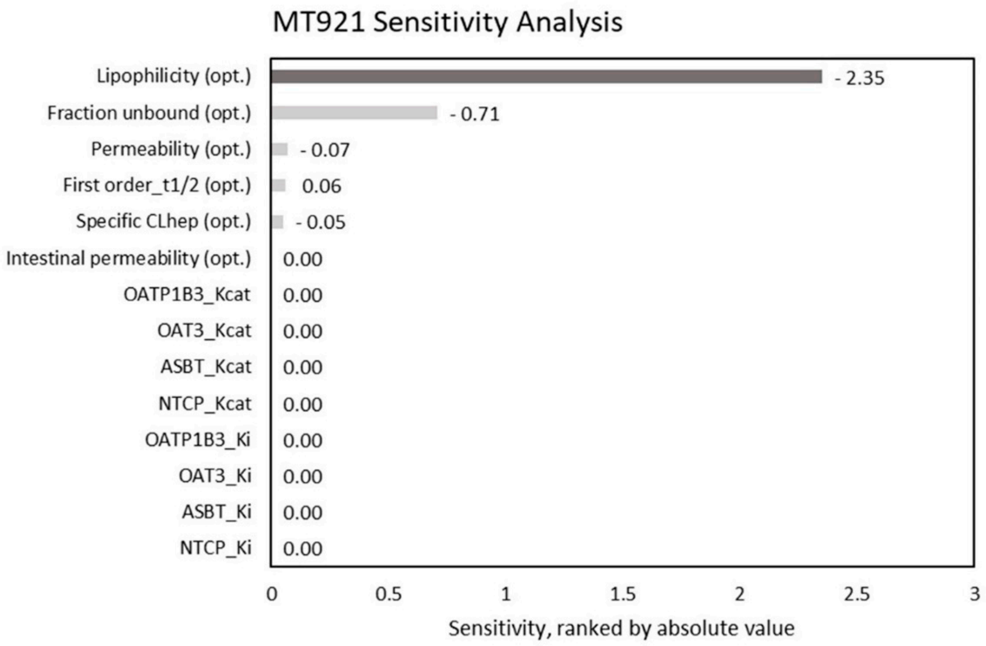


**Figure S7.** MT921 model sensitivity analysis. Sensitivity of each parameter used in final model. Sensitivity is measured by AUC change in MT921 150mg SC simulation. $K_{cat}$, catalytic rate constant; $K_i$, inhibition constant; $CL_{hep}$, total hepatic clearance; OATP1B3, organic anion transporter protein 1B3; OAT3, organic anion transporter 3; ASBT, apical sodium-dependent bile acid transporter; NTCP, $Na^+$-taurocholate co-transporting polypeptide.

## 6. PBPK Modeling of Amlodipine

### *6.1. Clinical Studies*

**Table S11.** Summary of clinical studies used for amlodipine model.

| Dose [mg] | Route | N | Age [years] | Weight [kg] | Height [cm] | Female [%] | Data set | Reference |
|---|---|---|---|---|---|---|---|---|
| 10 | po (tab, md) | 34 | 27.4 ± 2.9 | 67.3 ± 6.6 | - | 0 | test set | [16] |
| 10 | po (tab, sd) | 12 | 23-34 (26.8) | 61.7-86.9 (75.1) | - | 0 | test set | [17] |
| 5 | po (tab, sd) | 12 | 23-34 (26.8) | 61.7-86.9 (75.1) | - | 0 | test set | [17] |
| 10 | po (tab, sd) | 12 | 19-26 (21.4) | 54-75 (61.3) | 162-177 (171.2) | 0 | training set | [18] |
| 10 | po (tab, sd) | 12 | 25.8 ± 3.8 | 66.6 ± 6.3 | - | 0 | test set | [19] |
| 5 | po (tab, sd) | 6 | 22.5 ± 1.4 | 68.3 ± 4.6 | 175. ± 4.3 | - | training set | [20] |

| | | | | | | | | |
|---|---|---|---|---|---|---|---|---|
| 10 | po (tab, sd) | 6 | 24 ± 3.5 | 64.4 ± 6.7 | 174.7 ± 5.5 | - | training set | [20] |
| 10 | po (tab, md) | 20 | 31± 8 | 69.6 ± 8.1 | 175.5 ± 5 | - | test set | [21] |
| 10 | po (tab, sd) | 18 | 23.8 ± 2.6 | 73.9 ± 8 | 176.7 ± 6.2 | 0 | training set | [22] |
| 10 | po (tab, sd) | 18 | 22.7 ± 1.4 | 59.5 ± 5.6 | 163.4 ± 6 | 100 | test set | [22] |
| 5 | po (tab, sd) | 30 | - | - | - | - | test set | [23] |
| 10 | po (tab, sd) | 20 | - | - | - | - | test set | [24] |
| 5 | po (tab, sd) | 12 | 21-38 (26) | 67-92 (76) | 170-194 (182) | 0 | test set | [25] |
| 10 | po (tab, sd) | - | 26.8 ± 5.9 | 67.9 ± 8.2 | 174.2 ± 4.8 | - | test set | [26] |
| 5 | po (tab, sd) | 24 | 30.8 ± 9.4 | 61.1 ± 8.4 | 165.5 ± 6.7 | 33.3 | test set | [27] |
| 5 | po (tab, sd) | 24 | 33.0 ± 9.1 | 61.3 ± 6.0 | 164.5 ± 6.1 | 33.3 | training set | [27] |
| 2.5 | po (tab, sd) | 24 | 20-45 | - | - | - | test set | [28] |
| 10 | po (tab, sd) | 20 | 19-32 (23.7) | 60-90 (72) | - | - | training set | [29] |
| 5 | po (tab, sd) | 18 | 20-25 (22.3) | 57-77 (67.9) | - | - | training set | [30] |

po, per oral; tab, tablet; sd, single dose; md, multiple dose; If there are no demographic data, Asian [31], Japanese (2015), European [32] data in PK-sim database are used.

## *6.2. Drug-Dependent Parameters*

**Table S12.** Drug-dependent parameters of the amlodipine PBPK model.

| Parameter | Value | Unit | Source | Reference |
|---|---|---|---|---|
| MW | 408.9 | g/mol | - | - |
| Log P | 3.43 | Log units | Literature | [33] |
| Fraction unbound | 0.07 | - | Literature | [33] |
| Solubility | 75.3 | mg/l | - | - |
| Formulation | Weibull | | Optimized | - |
| Total $CL_{hep}$ | 0.39 | 1/h | | [34] |
| ASBT Ki | 42.10 | | | [35] |
| GFR | 1 | - | - | - |
| Partition coefficients | Rogers and Rowland | - | Calculated | [36,37] |
| Cellular perm. | PK sim standard | - | Calculated | [15] |
| Specific organ perm. | 7.09E-4 | cm/min | Optimized | - |
| Specific intest. perm. | 3.84E-5 | cm/min | Optimized | - |

MW, molecular weight; Log P, lipophilicity; $CL_{hep}$, hepatic clearance; ASBT, apical sodium dependent bile acid transporter; GFR, glomerular filtration rat; intest., intestinal; perm., permeability.

## *6.3. Profile*

### 6.3.1. Semilogarithmic Plots – Plasma – Population Predictions

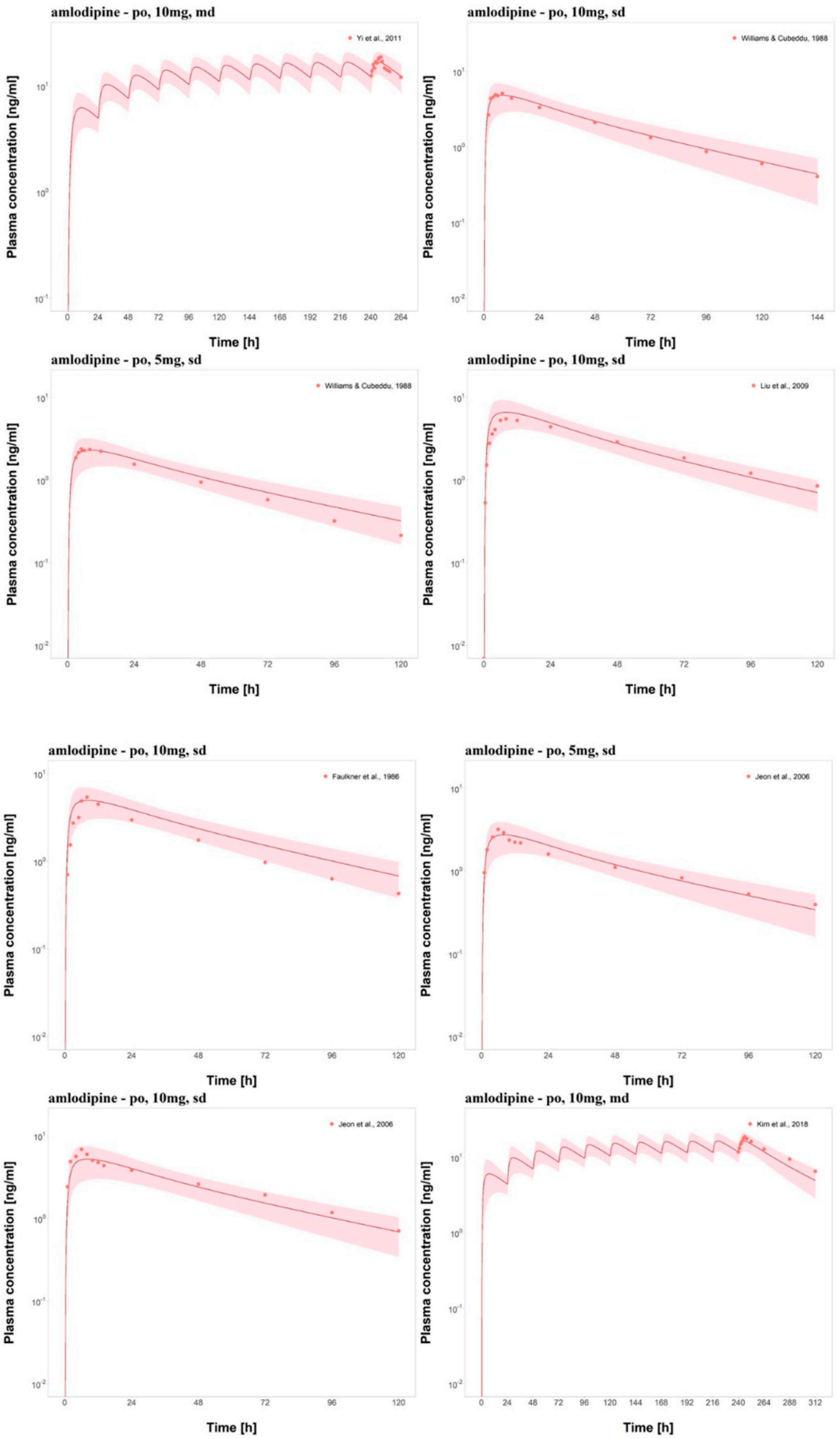


**Figure S8.** Amlodipine population plasma concentration-time profiles (semilogarithmic). Population predicted of MT921 plasma concentration-time profiles compared to observed data. Dot, observed data; Line, simulation arithmetic mean; shaded area, 95% confidence intervals of median predicted concentration.

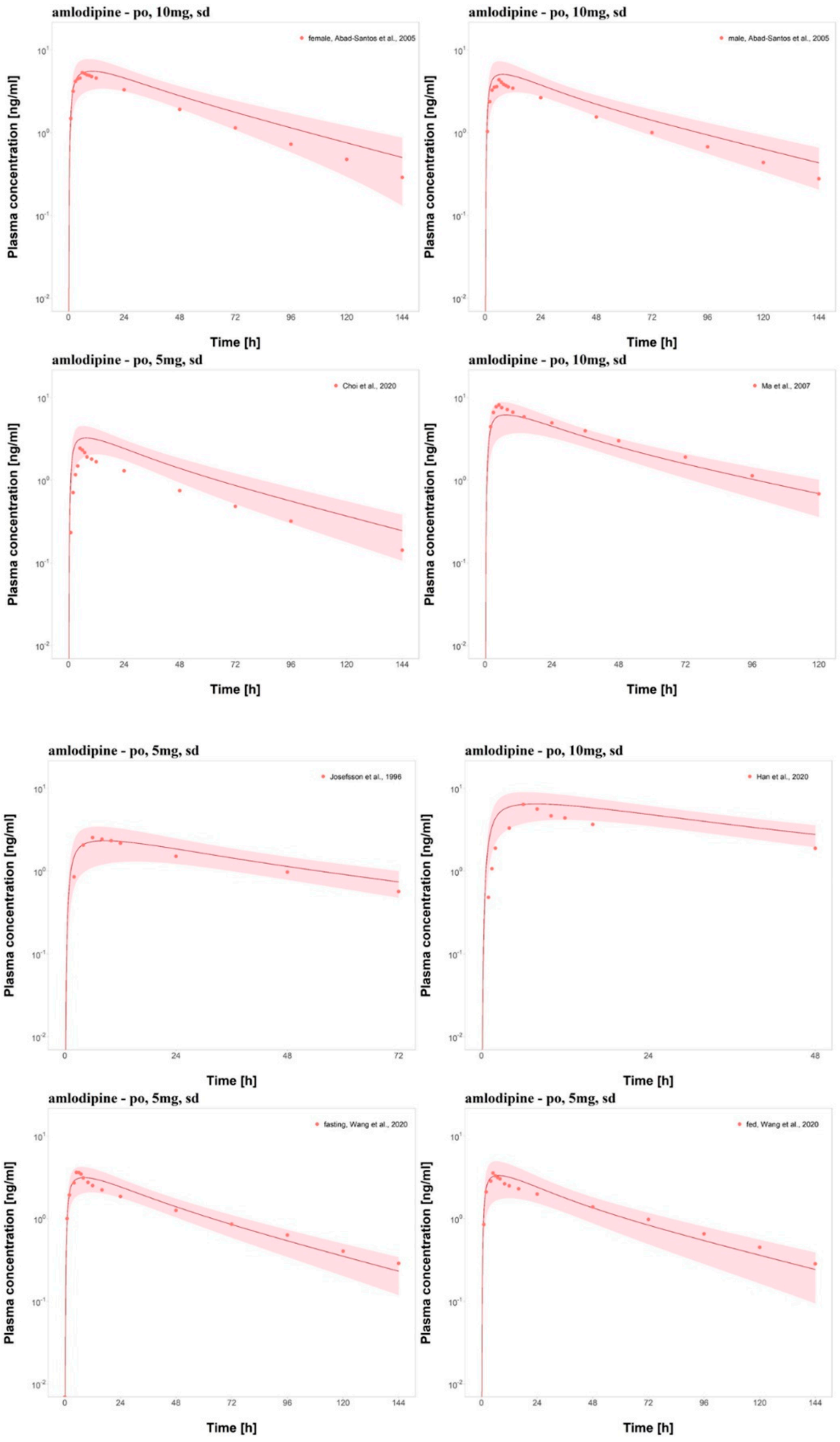


**Figure S8.** Amlodipine population plasma concentration-time profiles (semilogarithmic). Population predicted of MT921 plasma concentration-time profiles compared to observed data. Dot, observed data; Line, simulation arithmetic mean; shaded area, 95% confidence intervals of median predicted concentration.

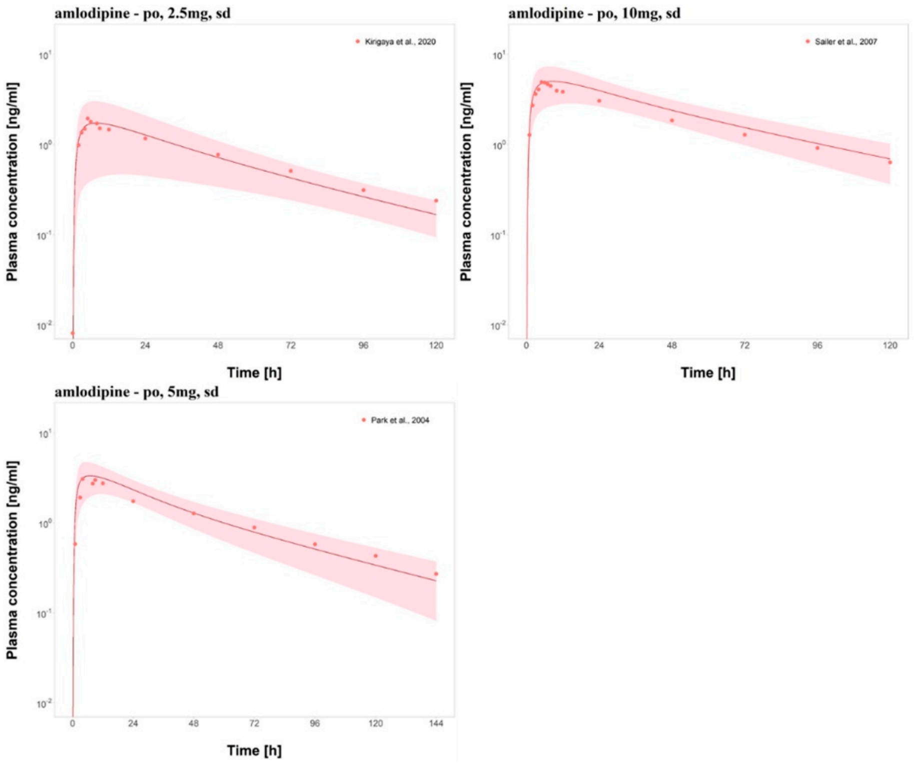


**Figure S8.** Amlodipine population plasma concentration-time profiles (semilogarithmic). Population predicted of MT921 plasma concentration-time profiles compared to observed data. Dot, observed data; Line, simulation arithmetic mean; shaded area, 95% confidence intervals of median predicted concentration.

6.3.2. Linear Plots – Plasma – Population Predictions

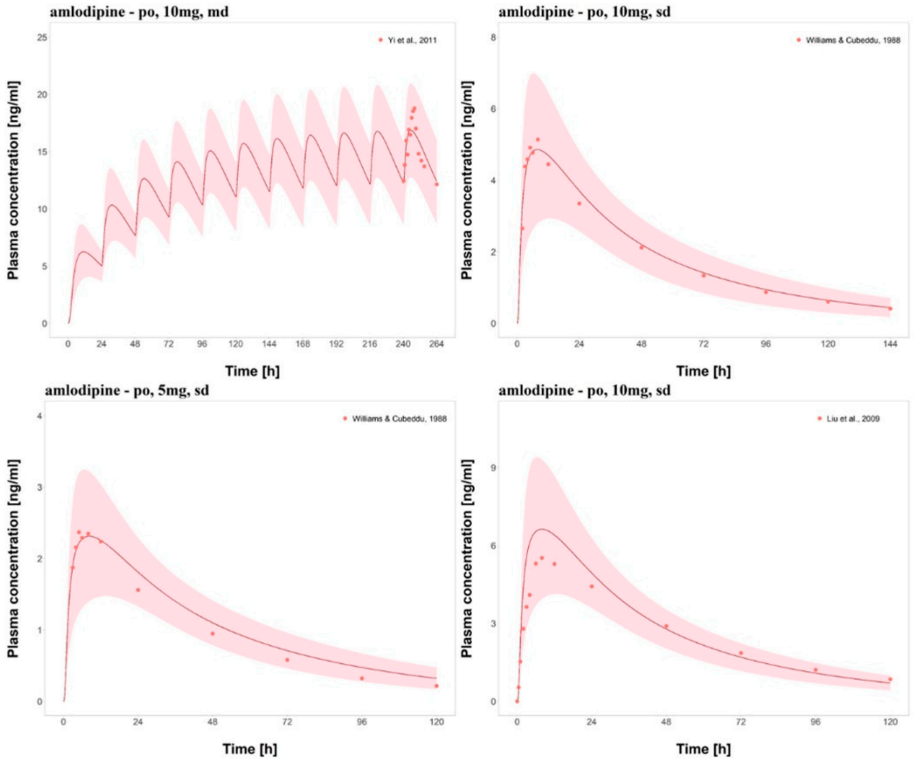

**Figure S9.** Amlodipine plasma concentration-time profiles (linear). Population predictions of amlodipine plasma concentration-time profiles compared to observed data. Dot, observed data; Line, simulation arithmetic mean; shaded area, 95% confidence intervals of median predicted concentration.

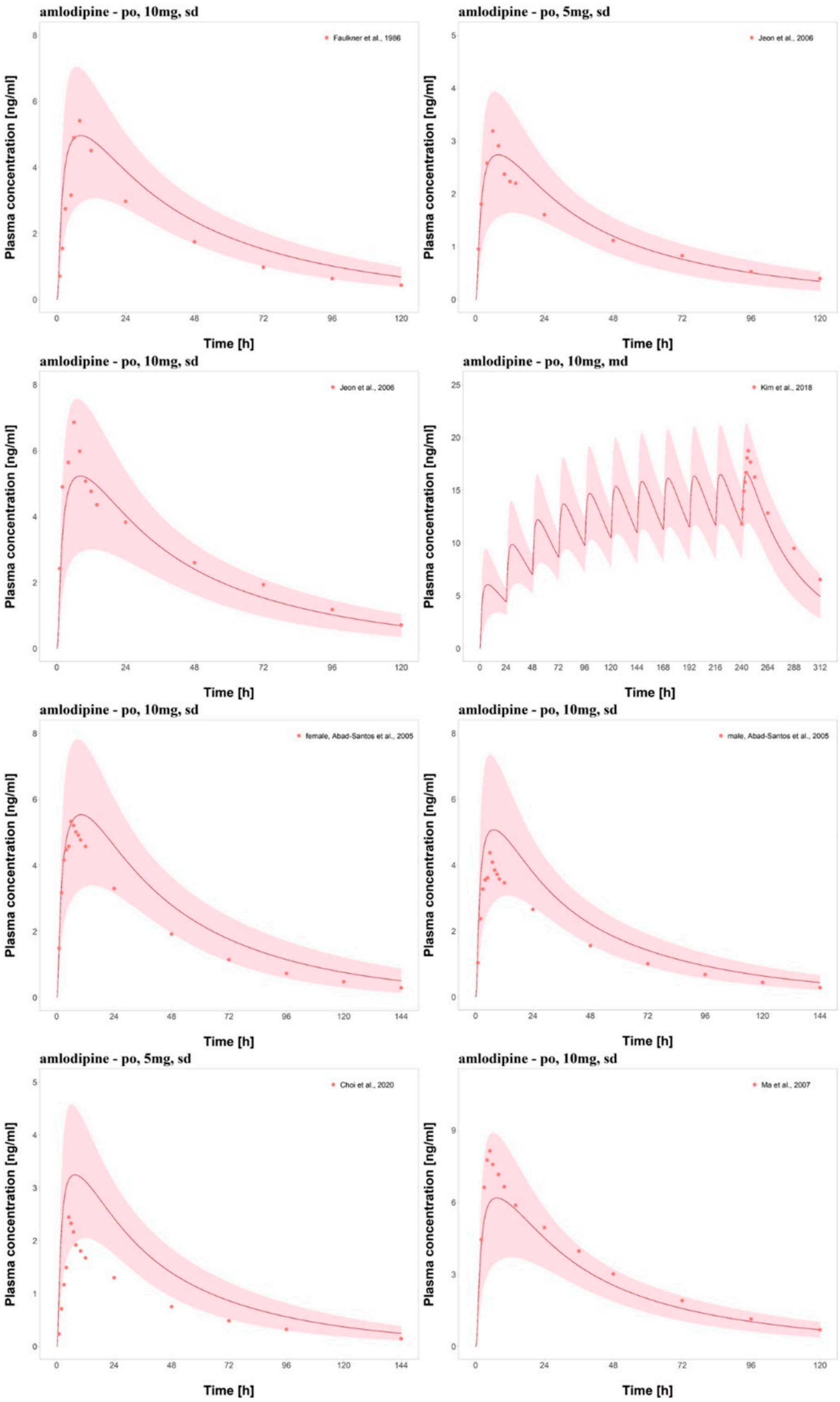


**Figure S9.** Amlodipine plasma concentration-time profiles (linear). Population predictions of amlodipine plasma concentration-time profiles compared to observed data. Dot, observed data; Line, simulation arithmetic mean; shaded area, 95% confidence intervals of median predicted concentration.

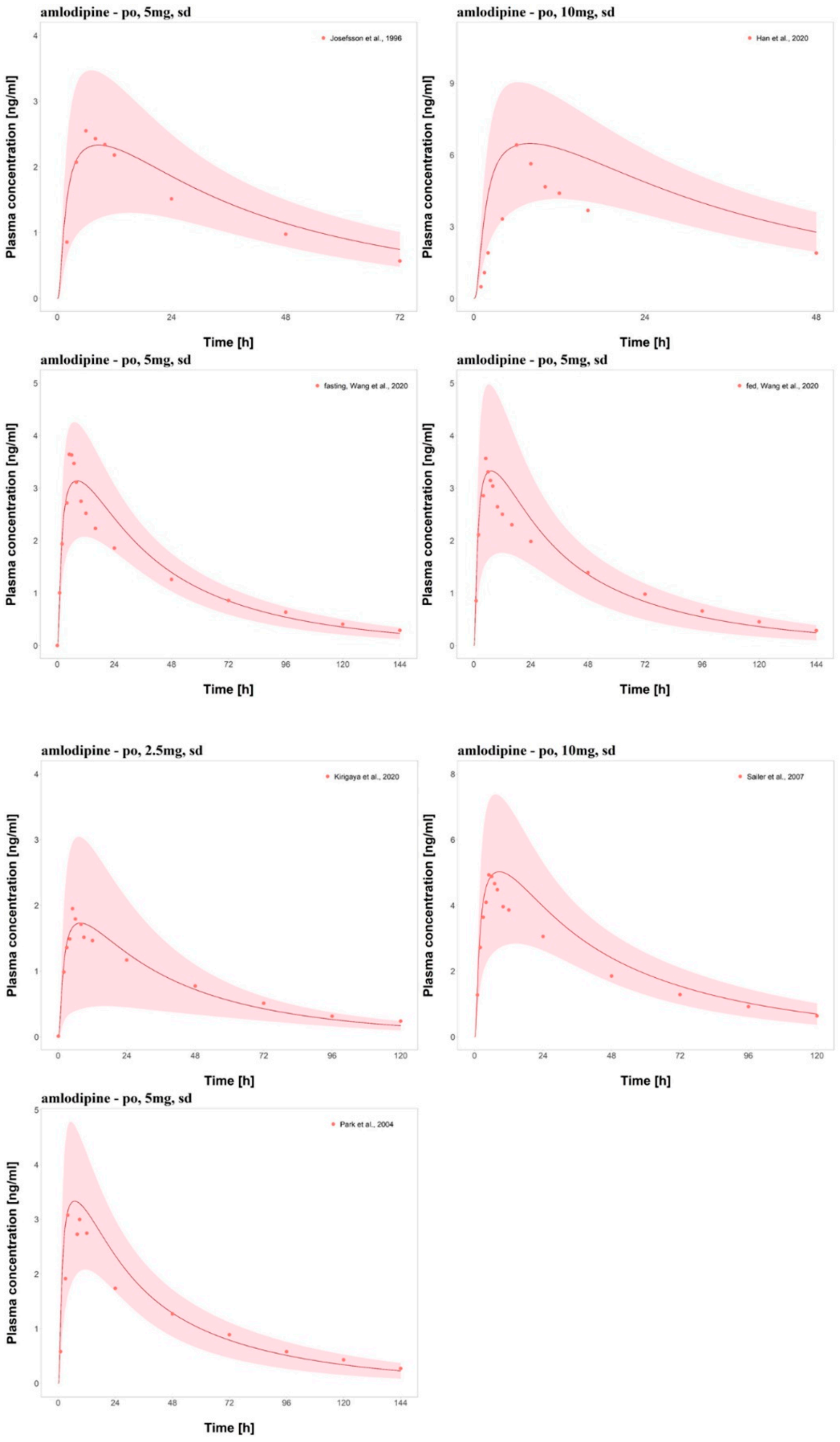


**Figure S9.** Amlodipine plasma concentration-time profiles (linear). Population predictions of amlodipine plasma concentration-time profiles compared to observed data. Dot, observed data; Line, simulation arithmetic mean; shaded area, 95% confidence intervals of median predicted concentration.

### *6.4. Amlodipine PBPK Model Evaluation*

#### 6.4.1. Plasma Concentration Goodness-of-Fit Plot

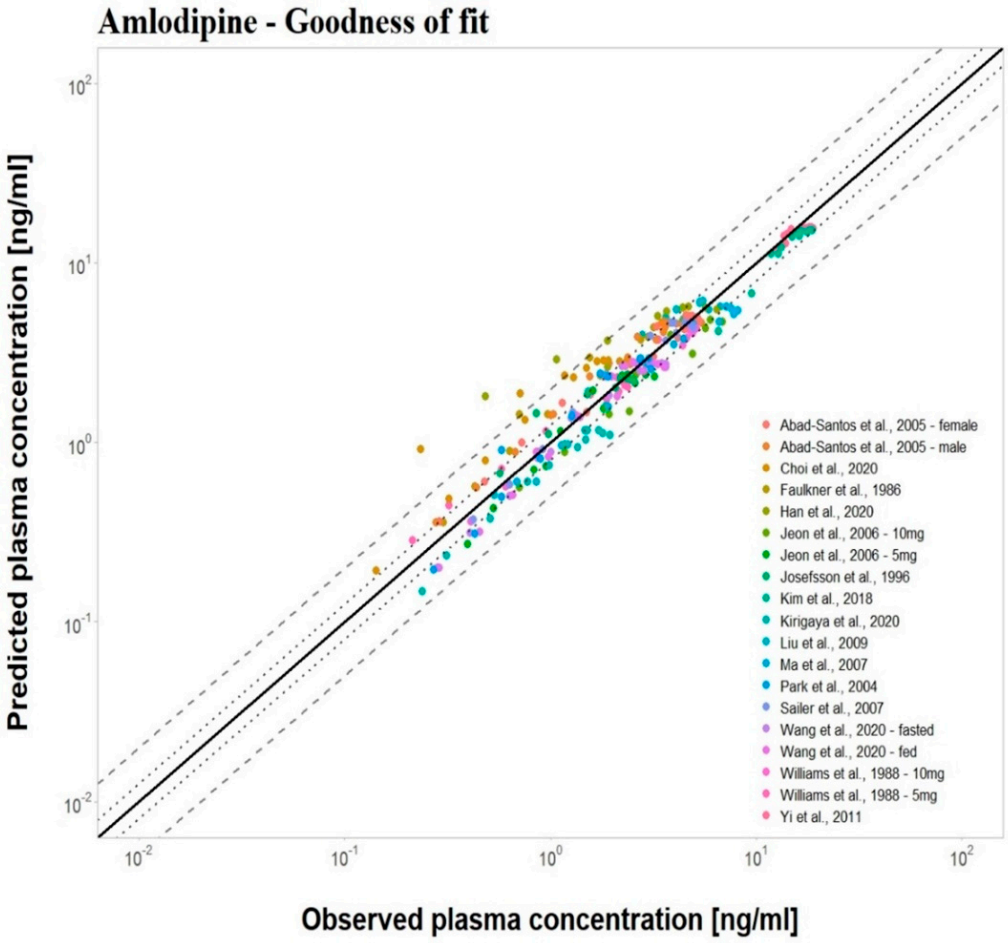


**Figure S10.** Goodness-of-fit plot of amlodipine. Predicted plasma concentration versus observed plasma concentration was compared in all clinical studies. The solid line means the line of identity. The dot lines are 1.25-fold, the dash lines are 2-fold deviation.

### 6.4.2. Mean Relative Deviation (MRD) of Predicted Plasma Concentrations

**Table S13.** MRD values of predicted amlodipine plasma concentrations.

| Route | Dose [mg] | MRD | Reference |
|---|---|---|---|
| po (tab), md | 10 | 1.09 | [16] |
| po (tab), sd | 10 | 1.13 | [17] |
| po (tab), sd | 5 | 1.19 | [17] |
| po (tab), sd | 10 | 1.24 | [18] |
| po (tab), sd | 10 | 1.44 | [19] |
| po (tab), sd | 5 | 1.21 | [20] |
| po (tab), sd | 10 | 1.30 | [20] |
| po (tab), md | 10 | 1.21 | [21] |
| po (tab), sd | 10 | 1.21 | [22] |
| po (tab), sd | 10 | 1.29 | [22] |
| po (tab), sd | 5 | 1.84 | [23] |
| po (tab), sd | 10 | 1.25 | [24] |
| po (tab), sd | 5 | 1.23 | [25] |

| | | | |
|---|---|---|---|
| po (tab), sd | 10 | 1.84 | [26] |
| po (tab), sd | 5 | 1.21 | [27] |
| po (tab), sd | 5 | 1.21 | [27] |
| po (tab), sd | 2.5 | 1.41 | [28] |
| po (tab), sd | 10 | 1.14 | [29] |
| po (tab), sd | 5 | 1.25 | [30] |
| **MRD** | | **1.29 (1.09 – 1.84)** | |
| | | **19/19 with MRD ≤ 2** | |

po, per oral; tab, tablet; md, multiple dose; sd, single dose; MRD, mean relative deviation.

#### 6.4.3. Predicted and Observed Pharmacokinetic (PK) Parameter Values with Mean GMFE Values

**Table S14.** Observed and predicted amlodipine AUC and $C_{max}$ values with mean GMFE values .

| Route | Dose [mg] | AUC | | | $C_{max}$ | | | Reference |
|---|---|---|---|---|---|---|---|---|
| | | Pred [ng*h/ml] | Obs [ng*h/ml] | Pred/Obs | Pred [ng/ml] | Obs [ng/ml] | Pred/Obs | |
| po (tab), md | 10 | 364.27 | 352.93 | 1.03 | 15.95 | 18.77 | 0.85 | [16] |
| po (tab), sd | 10 | 264.36 | 256.51 | 1.03 | 4.41 | 5.13 | 0.85 | [17] |
| po (tab), sd | 5 | 126.67 | 108.78 | 1.16 | 2.20 | 2.36 | 0.93 | [17] |
| po (tab), sd | 10 | 319.65 | 316.31 | 1.01 | 6.19 | 5.51 | 1.12 | [18] |
| po (tab), sd | 10 | 272.69 | 217.94 | 1.25 | 4.69 | 5.40 | 0.86 | [19] |
| po (tab), sd | 5 | 130.52 | 134.09 | 0.97 | 2.39 | 3.18 | 0.75 | [20] |
| po (tab), sd | 10 | 266.79 | 301.82 | 0.88 | 4.86 | 6.85 | 0.71 | [20] |
| po (tab), md | 10 | 882.33 | 832.27 | 1.06 | 15.30 | 18.73 | 0.81 | [21] |
| po (tab), sd | 10 | 307.26 | 241.79 | 1.27 | 5.12 | 5.32 | 0.96 | [22] |
| po (tab), sd | 10 | 271.99 | 197.94 | 1.37 | 4.70 | 4.37 | 1.07 | [22] |
| po (tab), sd | 5 | 158.67 | 96.48 | 1.64 | 2.88 | 2.44 | 1.18 | [23] |
| po (tab), sd | 10 | 305.65 | 248.67 | 1.22 | 5.77 | 8.13 | 0.71 | [24] |
| po (tab), sd | 5 | 104.06 | 92.30 | 1.12 | 2.27 | 2.54 | 0.89 | [25] |
| po (tab), sd | 10 | 205.47 | 152.68 | 1.34 | 5.72 | 6.41 | 0.89 | [26] |
| po (tab), sd | 5 | 161.44 | 167.48 | 0.96 | 2.83 | 3.56 | 0.79 | [27] |
| po (tab), sd | 5 | 160.49 | 158.67 | 1.01 | 2.83 | 3.64 | 0.77 | [27] |
| po (tab), sd | 2.5 | 66.86 | 87.62 | 0.76 | 1.18 | 1.94 | 0.61 | [28] |
| po (tab), sd | 10 | 276.74 | 239.50 | 1.15 | 4.70 | 4.92 | 0.95 | [29] |
| po (tab), sd | 5 | 164.06 | 155.04 | 1.05 | 2.96 | 3.07 | 0.96 | [30] |
| **GMFE** | | | | **1.16 (1.01-1.64)** | | | **1.19 (1.04-1.64)** | |
| | | | | **19/19 GMFE ≤ 2** | | | **19/19 GMFE ≤ 2** | |

AUC, area under the curve the plasma concentration-time curve; $C_{max}$, peak plasma concentration; GMFE, geometric mean fold error; sd, single dose; md, multiple dose; Pred, predicted; Obs, observed; route, route of administration of drug; tab, tablet; po, per oral.

#### 6.4.4. Sensitivity Analysis

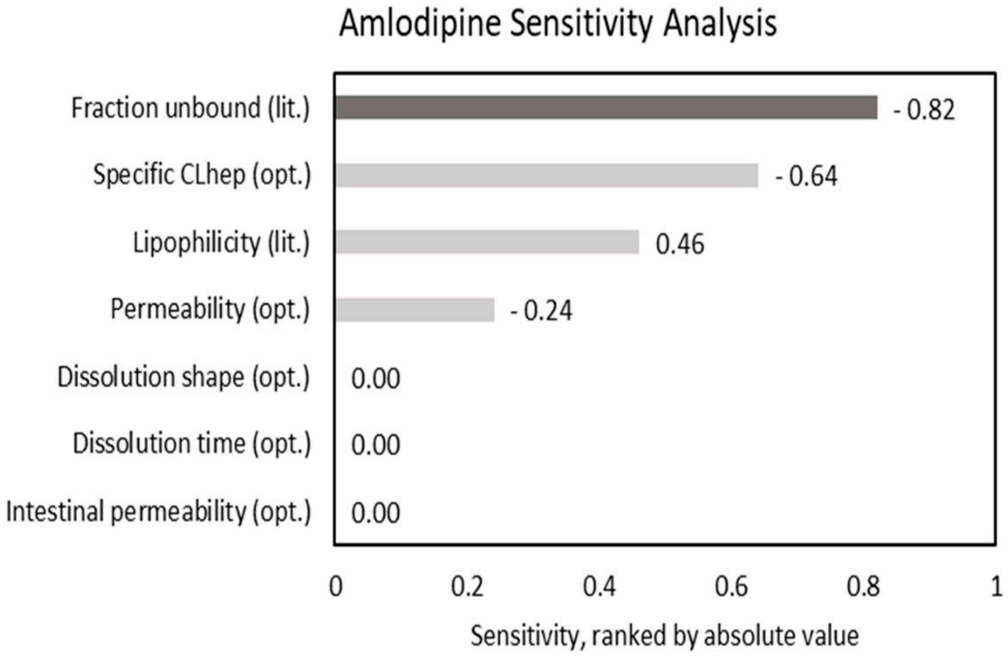


**Figure S11.** Amlodipine model sensitivity analysis. Sensitivity of each parameter used in final model. Sensitivity is measured by AUC change in amlodipine 10mg per oral simulation. Opt., optimized value; lit., literature value; $CL_{hep}$, total hepatic clearance.

## 7. Prediction of DDI Potential

*7.1 Profile*

7.1.1 Linear Graph – Comparing MT921 Alone Concentration and Population Simulation of MT921 Co-Administration.

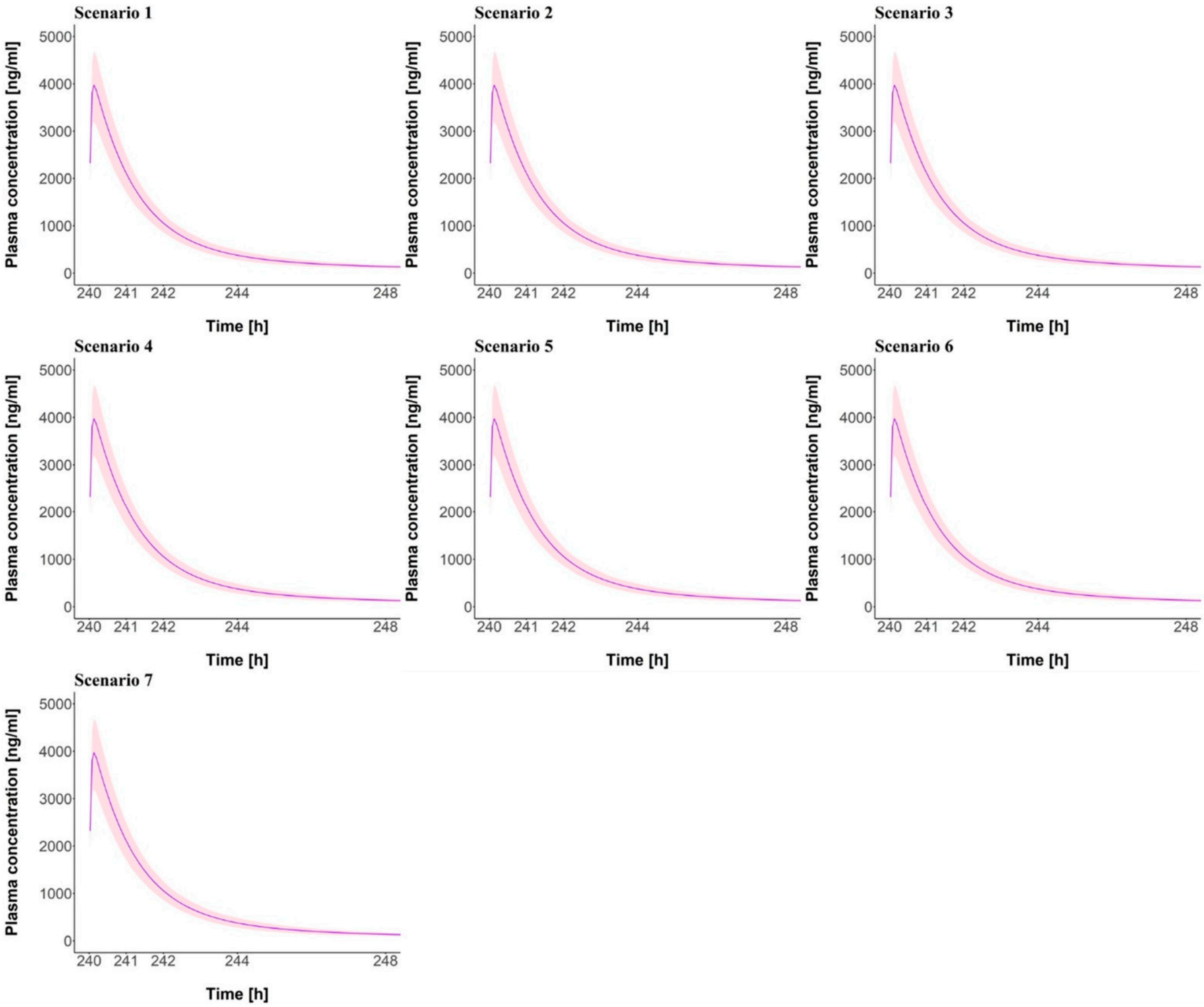


**Figure S12.** Linear graph comparing plasma concentration of MT921 administered alone and co-administered. Chronic disease drugs are administered during 9 days, once a day. At 10 days, 150mg of MT921 was co-administered with chronic disease drugs. After administration, MT921 concentration was predicted. Purple line is MT921 alone population plasma concentration. Pink shade area is 5 to 95% range of MT921 co-administration population plasma concentration.

### 7.1.2 Semilogarithmic Graph – Comparing MT921 Alone Concentration and Population Simulation of MT921 Co-Administration.

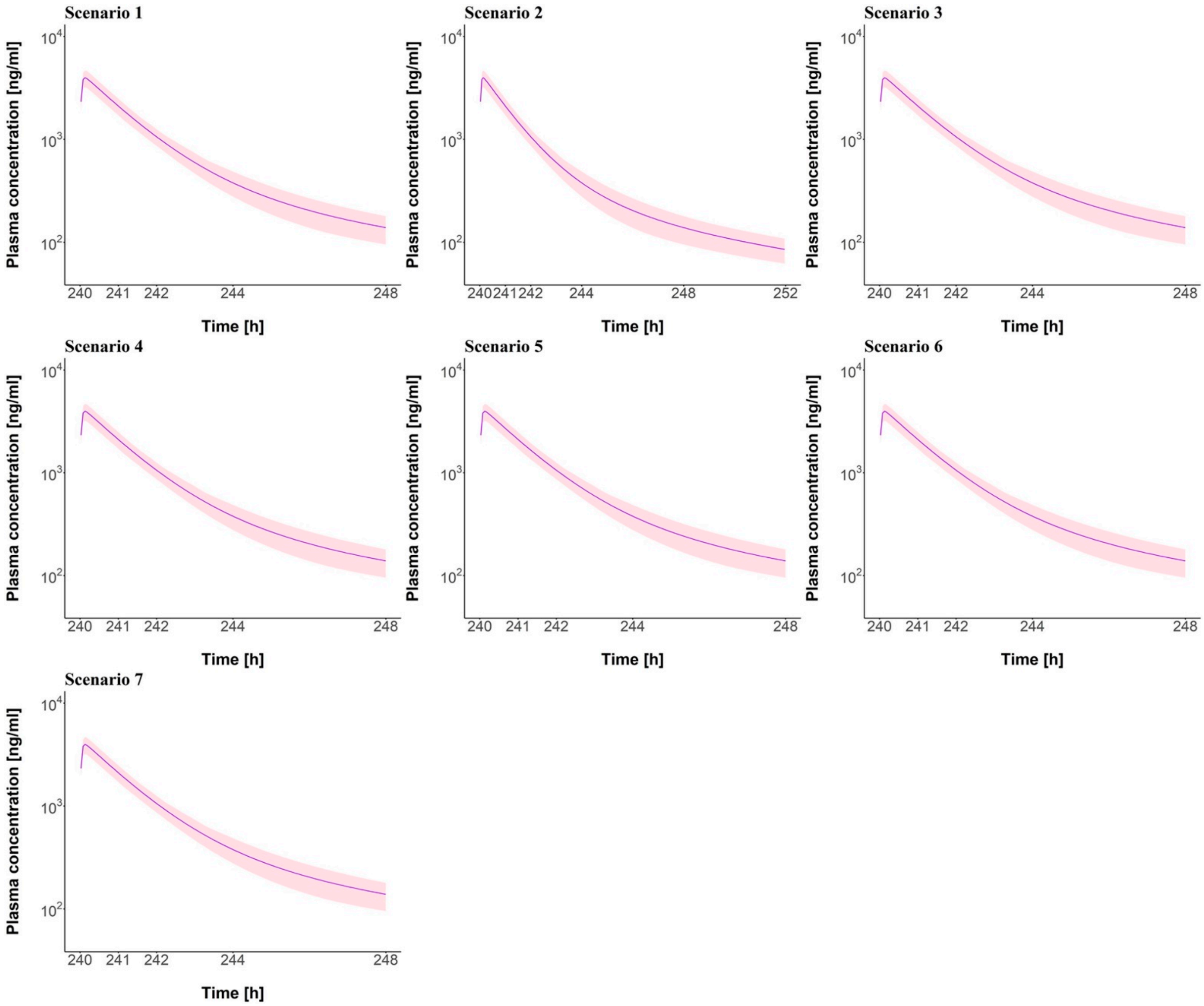


**Figure S13.** Semilogarithmic graph comparing plasma concentration of MT921 administered alone and co-administered. Chronic disease drugs are administered during 9 days, once a day. At 10 days, 150mg of MT921 was co-administered with chronic disease drugs. After administration, MT921 concentration was predicted. Purple line is MT921 alone population plasma concentration. Pink shade area is 5 to 95% range of MT921 co-administration population plasma concentration.

## References


1. Kim, M.-J.; Kim, H.; Cha, I.-J.; Park, J.-S.; Shon, J.-H.; Liu, K.-H; Shin, J.-G. High-throughput screening of inhibitory potential of nine cytochrome P450 enzymes *in vitro* using liquid chromatography/tandem mass spectrometry. *Rapid Commun. Mass Spectrom.* **2005**, *19*, 2651–2658, doi: 10.1002/rcm.2110.
2. Seo, K.-A.; Kim, H.-J; Jeong, E. S.; Abdalla, N.; Choi, C.-S.; Kim, D.-H; Shin, J.-G. In Vitro Assay of Six UDP-glucuronosyltransferase Isoforms in Human Liver Microsomes, Using Cocktails of Probe Substrates and Liquid Chromatography-Tandem Mass Spectrometry. *Drug Metab Dispos.* **2014**, *42*, 1803–1810, doi: 10.1124/dmd.114.058818.
3. Rautio, J.; Humphreys, J.E.; Webster, L.O.; Balakrishnan, A.; Keogh, J.P.; Kunta, J.R; Serabjit-Singh, C.J; Polli, J.W. IN VITRO P-GLYCOPROTEIN INHIBITION ASSAYS FOR ASSESSMENT OF CLINICAL DRUG INTERACTION POTENTIAL OF NEW DRUG CANDICATES: A RECOMMENDATION FOR PROBE SUBSTRATES. *Drug Metab Dispos.* **2006**, *34*, 786–792, doi: 10.1124/dmd.105.008615.
4. Xin, H.-W.; Schwab, M.; Klotz, U. Transport studies with 5-aminosalicylate. *Eur J Clin Pharmacol.* **2006**, *62*, 871–875, doi: 10.1007/s00228-006-0182-3.
5. Center for Drug Evaluation and Research (CDER). *Guidance for Industry: In Vitro Drug Interaction Studies-Cytochrome P450 Enzyme- and Transporter-Mediated Drug Interactions*; Food and Drug Administration: Silver Spring, MD, USA, 2020. Available online: http://www.fda.gov/media/134582/download (accessed on 10 January 2021).
6. Satoh, H.; Yamashita, F.; Tsujimoto, M.; Murakami, H.; Koyabu, N.; Ohtani, H.; Sawada, Y. Citrus juices inhibit the function of human organic anion-transporting polypeptide OATP-B. *Mol. Pharm.* **2005**, *33*, 518–523, doi: 10.1124/dmd.104.002337.
7. Ueo, H.; Motohashi, H.; Katsura, T.; Inui, K.-i. Human organic anion transporter hOAT3 is a potent transporter of cephalosporin antibiotics, in comparison with hOAT1. *Biochem. Pharmacol.* **2005**, *70*, 1104–1113, doi: 10.1016/j.bcp.2005.06.024.
8. Ahlin, G.; Chen, L.; Lazorova, L.; Chen, Y.; Ianculescu, A.G.; Davis, R.L.; Giacomini, K.M.; Artursson, P. Genotype-dependent effects of inhibitors of the organic cation transporter, OCT1: predictions of metformin interactions. *Pharmacogenomics. J.* **2011**, *11*, 400–411, doi: 10.1038/tpj.2010.54.
9. Kimura, N.; Masuda, S.; Tanihara, Y.; Ueo, H.; Okuda, M.; Katsura, Y.; Inu, K.-i. Metformin is a superior substrate for renal organic cation transporter OCT2 rather than hepatic OCT1. *Drug. Metab. Pharmacokinet.* **2005**, *20*, 379–386, doi: 10.2133/dmpk.20.379.
10. *Instructions for Use of Products: CellTiter-Glo® Luminescent Cell Viability Assay*; Promega Corporation: Madison, WI, USA, Revised 3/15. Available online: https://www.promega.com/-/media/files/resources/protocols/technical-bulletins/0/celltiter-glo-luminescent-cell-viability-assay-protocol.pdf (accessed on 10 January 2021).
11. Roda, A.; Minutello, A.; Angellotti, M.; Fini, A. Bile Acid Structure-Activity Relationship: Evaluation of Bile Acid Lipophilicity Using 1-Octanol/Water Partition Coefficient and Reverse Phase HPLC. *J. Lipid Res.* **1990**, *31*, 1433–1443, doi:10.1016/S0022-2275(20)42614-8.
12. Salvioli, G.; Lugli, R.; Pradelli, J.M.; Gigliotti, G. Bile Acid Binding in Plasma: The Importance of Lipoproteins. *FEBS Lett.* **1985**, *187*, 272–276, doi:10.1016/0014-5793(85)81257-6.
13. Engelking, L.R.; Barnes, S.; Dasher, C.A.; Naftel, D.C.; Hirschowitz, B.I. Radiolabelled Bile Acid Clearance in Control Subjects and Patients with Liver Disease. *Clin. Sci.* **1979**, *57*, 499–508, doi:10.1042/cs0570499.
14. Schmitt, W. General Approach for the Calculation of Tissue to Plasma Partition Coefficients. *Toxicol In Vitro* **2008**, *22*, 457–467, doi:10.1016/j.tiv.2007.09.010.
15. Open Systems Pharmacology Suite Community. Open Systems Pharmacology Suite Manual. Available online: https://docs.open-systems-pharmacology.org/shared-tools-and-example-workflows/sensitivity-analysis (accessed on 26 May 2021).
16. Yi, S.; Kim, T.-E.; Yoon, S.H.; Cho, J.-Y.; Shin, S.-G.; Jang, I.-J.; Yu, K.-S. Pharmacokinetic Interaction of Fimasartan, a New Angiotensin II Receptor Antagonist, with Amlodipine in Healthy Volunteers. *J Cardiovasc Pharmacol* **2011**, *57*, 682–689, doi:10.1097/FJC.0b013e31821795d0.
17. Williams, D.M.; Cubeddu, L.X. Amlodipine Pharmacokinetics in Healthy Volunteers. *J Clin Pharmacol* **1988**, *28*, 990–994, doi:10.1002/j.1552-4604.1988.tb03119.x.
18. Liu, Y.; Jia, J.; Liu, G.; Li, S.; Lu, C.; Liu, Y.; Yu, C. Pharmacokinetics and Bioequivalence Evaluation of Two Formulations of 10-Mg Amlodipine Besylate: An Open-Label, Single-Dose, Randomized, Two-Way Crossover Study in Healthy Chinese Male Volunteers. *Clin Ther* **2009**, *31*, 777–783, doi:10.1016/j.clinthera.2009.04.013.
19. Faulkner, J.K.; McGibney, D.; Chasseaud, L.F.; Perry, J.L.; Taylor, I.W. The Pharmacokinetics of Amlodipine in Healthy Volunteers after Single Intravenous and Oral Doses and after 14 Repeated Oral Doses given Once Daily. *Br J Clin Pharmacol* **1986**, *22*, 21–25, doi:10.1111/j.1365-2125.1986.tb02874.x.
20. Jeon, J.Y.; Kim, J.R.; Chung, J.Y.; Cho, J.-Y.; Yu, K.-S.; Jang, I.-J.; Shin, S.G. Pharmacokinetic and Pharmacodynamic Comparative Study of Amlodipine Adipate and Amlodipine Besylate. *J Korean Soc Clin Pharmacol Ther* **2006**, *14*, 106–115, doi:10.12793/jkscpt.2006.14.2.106.
21. Kim, J.-R.; Kim, S.; Huh, W.; Ko, J.-W. No Pharmacokinetic Interactions between Candesartan and Amlodipine Following Multiple Oral Administrations in Healthy Subjects. *Drug Des Devel Ther* **2018**, *12*, 2475–2483, doi:10.2147/DDDT.S172568.

22. Abad-Santos, F.; Novalbos, J.; Gálvez-Múgica, M.-A.; Gallego-Sandín, S.; Almeida, S.; Vallée, F.; García, A.G. Assessment of Sex Differences in Pharmacokinetics and Pharmacodynamics of Amlodipine in a Bioequivalence Study. *Pharmacol Res* **2005**, *51*, 445–452, doi:10.1016/j.phrs.2004.11.006.
23. Choi, S.; Jeon, S.; Yim, D.-S.; Han, S. Contribution of Trough Concentration Data in the Evaluation of Multiple-Dose Pharmacokinetics for Drugs with Delayed Distributional Equilibrium and Long Half-Life. *Drug Des Devel Ther* **2020**, *14*, 811–821, doi:10.2147/DDDT.S236701.
24. Ma, Y.; Qin, F.; Sun, X.; Lu, X.; Li, F. Determination and Pharmacokinetic Study of Amlodipine in Human Plasma by Ultra Performance Liquid Chromatography-Electrospray Ionization Mass Spectrometry. *J Pharm Biomed Anal* **2007**, *43*, 1540–1545, doi:10.1016/j.jpba.2006.11.015.
25. Josefsson, M.; Zackrisson, A.L.; Ahlner, J. Effect of Grapefruit Juice on the Pharmacokinetics of Amlodipine in Healthy Volunteers. *Eur J Clin Pharmacol* **1996**, *51*, 189–193, doi:10.1007/s002280050183.
26. Han, J.M.; Yee, J.; Chung, J.E.; Lee, K.E.; Park, K.; Gwak, H.S. Effects of Cytochrome P450 Oxidoreductase Genotypes on the Pharmacokinetics of Amlodipine in Healthy Korean Subjects. *Mol Genet Genomic Med* **2020**, *8*, e1201, doi:10.1002/mgg3.1201.
27. Wang, T.; Wang, Y.; Lin, S.; Fang, L.; Lou, S.; Zhao, D.; Zhu, J.; Yang, Q.; Wang, Y. Evaluation of Pharmacokinetics and Safety with Bioequivalence of Amlodipine in Healthy Chinese Volunteers: Bioequivalence Study Findings. *J Clin Lab Anal* **2020**, *34*, e23228, doi:10.1002/jcla.23228.
28. Kirigaya, Y.; Shiramoto, M.; Ishizuka, T.; Uchimaru, H.; Irie, S.; Kato, M.; Shimizu, T.; Nakatsu, T.; Nishikawa, Y.; Ishizuka, H. Pharmacokinetic Interactions of Esaxerenone with Amlodipine and Digoxin in Healthy Japanese Subjects. *BMC Pharmacol Toxicol* **2020**, *21*, 55, doi:10.1186/s40360-020-00423-4.
29. Sailer, R.; Arnold, P.; Erenmemişoğlu, A.; Martin, W.; Tamur, U.; Kanzik, I.; Hincal, A.A. Pharmacokinetics and Bioequivalence Study of a Generic Amlodipine Tablet Formulation in Healthy Male Volunteers. *Arzneimittelforschung* **2007**, *57*, 462–466, doi:10.1055/s-0031-1296632.
30. Park, J.-Y.; Kim, K.-A.; Lee, G.-S.; Park, P.-W.; Kim, S.-L.; Lee, Y.-S.; Lee, Y.-W.; Shin, E.-K. Randomized, Open-Label, Two-Period Crossover Comparison of the Pharmacokinetic and Pharmacodynamic Properties of Two Amlodipine Formulations in Healthy Adult Male Korean Subjects. Clin Ther 2004, 26, 715–723, doi:10.1016/s0149-2918(04)90071-9.
31. Tanaka, G. Anatomical and Physiological Characteristics for Asian Reference Man-Male and Female of Different Age : Tanaka Model-. *NIRS* **1996**.
32. Valentin, J. Basic Anatomical and Physiological Data for Use in Radiological Protection: Reference Values: ICRP Publication 89. *ICRP* **2002**, *32*, 1–277, doi:10.1016/S0146-6453(03)00002-2.
33. Zhou, D.; Bui, K.; Sostek, M.; Al-Huniti, N. Simulation and Prediction of the Drug-Drug Interaction Potential of Naloxegol by Physiologically Based Pharmacokinetic Modeling. CPT Pharmacometrics Syst Pharmacol 2016, 5, 250–257, doi:10.1002/psp4.12070.
34. Kadono, K.; Akabane, T.; Tabata, K.; Gato, K.; Terashita, S.; Teramura, T. Quantitative Prediction of Intestinal Metabolism in Humans from a Simplified Intestinal Availability Model and Empirical Scaling Factor. Drug Metab Dispos 2010, 38, 1230–1237, doi:10.1124/dmd.109.029322
35. Zheng, X.; Ekins, S.; Raufman, J.-P.; Polli, J.E. Computational Models for Drug Inhibition of the Human Apical Sodium-Dependent Bile Acid Transporter. Mol Pharm 2009, 6, 1591–1603, doi:10.1021/mp900163d.
36. Rodgers, T.; Leahy, D.; Rowland, M. Physiologically Based Pharmacokinetic Modeling 1: Predicting the Tissue Distribution of Moderate-to-Strong Bases. J Pharm Sci 2005, 94, 1259–1276, doi:10.1002/jps.20322.
37. Taylor, M.J.; Tanna, S.; Sahota, T. In Vivo Study of a Polymeric Glucose-Sensitive Insulin Delivery System Using a Rat Model. J Pharm Sci 2010, 99, 4215–4227, doi:10.1002/jps.22138.
